# ﻿Embioptera (Insecta) from Brazil: New species and a taxonomic update

**DOI:** 10.3897/zookeys.1088.72910

**Published:** 2022-03-10

**Authors:** Claudia Szumik, Verónica Pereyra, Victoria E. Goloboff Szumik, Paula Jéssica Costa-Pinto, María Laura Juárez

**Affiliations:** 1 Unidad Ejecutora Lillo, Consejo Nacional de Investigaciones Científicas y Técnicas, Fundación Miguel Lillo, Miguel Lillo 251, 4000, S. M. de Tucumán, Argentina Unidad Ejecutora Lillo Tucumán Argentina; 2 Facultad de Ciencias Naturales e Instituto Miguel Lillo, Universidad Nacional de Tucumán, Argentina Universidad Nacional de Tucumán Tucumán Argentina; 3 Instituto Nacional de Pesquisas da Amazônia, INPA, Manaus, Amazonas, Brazil Instituto Nacional de Pesquisas da Amazônia Amazonas Brazil; 4 Programa de Pós-Graduação em Entomologia, Departamento de Zoologia, Universidade Federal do Paraná, Curitiba, Paraná, Brazil Universidade Federal do Paraná Paraná Brazil

**Keywords:** Amazonia, Anisembiidae, Archembiidae, Clothodidae, Embiidina, neotropical, Teratembiidae, webspinners

## Abstract

Eight new species of Embioptera from Brazil are described, diagnosed, and illustrated. For Anisembiidae: *Chelicercaachilata* Szumik, Pereyra & Juárez, **sp. nov.**; *Saussurembiaborba* Szumik, Pereyra & Juárez, **sp. nov.** For Archembiidae: *Archembiaoruma* Szumik, **sp. nov.**; *Embolynthaoriximina* Szumik, Pereyra & Juárez, **sp. nov.**; *Pararhagadochirbonita* Szumik, Pereyra & Juárez, **sp. nov.**, *Pararhagadochirmarielleae* Szumik, Pereyra & Juárez, **sp. nov.**; *Pararhagadochirpara* Szumik, Pereyra & Juárez, **sp. nov.** For Clothodidae: *Chromatoclothodalanga* Szumik, Pereyra & Juárez, **sp. nov.** To clarify the higher classification of the Order and to have an accurate taxonomy, a species catalog and introduction to the four families present in Brazil is also detailed, including phylogenetic relationships, taxonomic actions, composition, distributions, and records maps. Herein, several taxonomic acts are proposed: (1) the synonymy of *Chelicerca* Ross (= *Dactylocerca* Ross **confirmed junior synonym**; = *Schizembia* Ross **syn nov.**; = *Pelorembia* Ross, **confirmed junior synonym**; = *Cryptembia* Ross, **syn. nov.**) and *Saussurembia* Davis (= *Stenembia* Ross, **syn. nov.**). (2) new status and delimitation for family Archembiidae Ross, **stat. rev.**; subfamily Archembiinae Ross, **stat. rev.**; subfamily Pachylembiinae**stat. rev.**; subfamily Scelembiinae**stat. rev.**, and their genera included. (3) *Diradiusunicolor* (Ross) (Teratembiidae) **comb. nov.**, and (4) new locality records for previously cited species in the region.

## ﻿Introduction

The biodiversity of Brazil harbors the highest number of insect species on Earth, reaching approximately 9% of the total known species, where many of them are endemic taxa (Rafael et al. 2012). However, unfortunately, the knowledge on the real biodiversity of Brazil can only be estimated and remains scant, even in protected areas (see e.g., Rafael et al. 2009; Diniz-Filho et al. 2010; Carvalho 2012; Oliveira et al. 2017). This is also the case of the Order Embioptera whose representativeness of species is very low if we consider the surface and the diversity of biomes present in the country (Rafael et al. 2012; Szumik 2012). Szumik (2012) recorded four of the five families of American embiids for Brazil, i.e., Archembiidae, Anisembiidae, Teratembiidae, and Clothodidae. These families were represented by 21 genera and 41 species, a number of species that should at least be triplicated. In the last years, only six new species were described for Brazil, one clothodid (Krolow and Valadares 2016) and five archembiids (Szumik et al. 2017; Salvatierra 2020; Costa-Pinto et al. 2021). In addition, new records for an archembiid and an anisembiid were reported for two states of Brazil (Teixeira et al. 2018a, b) as well as records of presence of the Order in studies of microhabitat diversity (Lopes Ferreira and Sousa Silva 2001; Sousa Silva et al. 2009).

Here, we describe eight new species from Brazil, most of them from the Amazonian region; we also add new records for previously cited species in the region. The classification used here follows Szumik et al. (2019), which is the most accurate analysis involving the total evidence to date. To have a better understanding of the higher classification of the Order, an introduction to the families present in Brazil is developed. The genera where the new species are included have a section with diagnosis, composition, distribution, and phylogenetic relationships. Finally, for the first time, a complete list of the Brazilian species is presented.

As Carvalho (2012) clearly argued, there are two major problems with Brazilian taxonomic studies: one is the excessive richness species of the country and the other, the reduced number of taxonomists. Hopefully, our observations will encourage young researchers to study the biodiversity of embiids from Brazil.

## ﻿Materials and methods

The material from the new species described here is deposited at Instituto Nacional de Pesquisas da Amazônia, Manaus, INPA collection, Museum of Zoology of the University of São Paulo, MZUSP collection, and Museum of Comparative Zoology, Harvard University, MCZ collection.

Many of the species known for Brazil were described by E.S. Ross (California Academy of Science) in his last monographs (e.g., Ross 2001, 2003), where the author stated that additional specimens of the described taxa were deposited in Brazilian Institutions like INPA and MZUSP; however, these specimens could not be found at those institutions. All the observations on the Brazilian specimens (e.g., species catalog, new records) are the result of the collection studies from several museums as well as the Ross collection (California Academy of Science). Museum collections acronyms:

**AMNH**American Museum of Natural History, New York, USA;

**CAS**California Academy of Science, Department of Entomology, Golden Gate Park, San Francisco, California, USA;

**FML**Fundación Miguel Lillo, Tucumán, Argentina;

**INPA**Instituto Nacional de Pesquisas da Amazônia, Manaus, Brazil;

**LABEI**Laboratório de Ecologia de Insetos, da Universidade Federal de Pelotas, Rio Grande do Sul, Brazil;

**MCZ**Museum of Comparative Zoology, Harvard University, Cambridge, Massachusetts, USA;

**MNHNP**Museum National d’Histoire Naturelle, Paris, France;

**MNHNPA**Museo Nacional de Historia Natural del Paraguay;

**MNRJ**Museo Nacional do Rio de Janeiro, Rio de Janeiro, Brazil;

**MZUSP**Museu de Zoologia, Universidade de São Paulo, São Paulo, Brazil;

**MZV**Museum and Institute of Zoology of the Polish Academy of Sciences, Warsaw, Poland;

**NHMUK**Natural History Museum, London, United Kingdom;

**USNM**National Museum of Natural History, Washington, USA;

**ZMB**Zoologisches Museum, Berlin, Germany.

Maps created using the free and open source QGIS ver 2.18 (http://www.qgis.org). All measurements are given in millimeters. Ocular ratio (**OR**) is defined as in Szumik (1991); features of the wing base union are presented in Szumik (1996); other abbreviations used are:

**Mm** mentum;

**Sm** submentum;

**10T** tenth tergite;

**10L** tenth left hemitergite;

**10R** tenth right hemitergite;

**10Lp1** caudal process of the 10L;

**10Rp1** caudal process of the 10R;

**10Rp2** anterior process of the 10R;

**Ep** epiproct;

**Lpp** left paraproct;

**Rpp** right paraproct;

**H** hypandrium or 9° sternite;

**Hp** process of H;

**LC1** basal left cercus;

**LC1dp** distal process of LC1;

**LC1bp** basal process of LC1;

**LC2** caudal left cercus.

## ﻿Results

### ﻿Family Anisembiidae Davis, 1940

Anisembiidae has an exclusively American distribution, and it is the most diverse group of the Order with 24 genera and more than 100 species assigned at different levels of subfamilies, tribes, or groups of species (Ross 2003). However, Ross’ classification does not have taxonomic and phylogenetic evidence to support it (Szumik 1996; Szumik et al. 2008; Miller et al. 2012), since some of the assignments were erroneously proposed, such as the creation of monotypic genera based on autapomorphic species or the definition of groups of species using geographical division as a criterion (Szumik et al. 2008, 2019). Although the monophyly of the family was supported in several phylogenetic analyses (e.g., Szumik 1996; Szumik et al. 2008; Miller et al. 2012), the inconsistency of some groupings is clear as well as the para/polyphyly of many of the proposed genera (e.g., *Pelorembia* Ross and *Dactylocerca* Ross; Szumik et al. 2008: 998). Currently, the family is under review but in this work we list the 19 valid genera, including several synonymies proposed here (see below for details): *Anisembia* Krauss, 1911; *Aporembia* Ross, 2003; *Brasilembia* Ross, 2003; *Bulbocerca* Ross, 1940; *Chelicerca* Ross, 1940 (= *Cryptembia* Ross, 2003; = *Dactylocerca*, Ross 1940; = *Pelorembia* Ross, 1984; = *Schizembia* Ross, 1944); *Chorisembia* Ross, 2003; *Ectyphocerca* Ross, 2003; *Exochosembia* Ross, 2003; *Glyphembia* Ross, 2003; *Isosembia* Ross, 2003; *Mesembia* Ross, 1940; *Microembia* Ross, 1944; *Oncosembia* Ross, 2003; *Phallosembia* Ross, 2003; *Platyembia* Ross, 2003; *Pogonembia* Ross, 2003; †*Poinarembia* Ross, 2003; *Saussurembia* Davis, 1940 (= *Stenembia* Ross, 1972); *Scolembia* Ross, 2003.

In this work, two new species of Anisembiidae are described, the synonymy of various genera is confirmed and, a complete list of known Brazilian species and new locality records are added. Consequently, in Brazil, Anisembiidae is represented by 13 species belonging to six genera (see details in Catalog).

#### ﻿Genus Chelicerca

Taxon classificationAnimaliaEmbiopteraAnisembiidae

Ross, 1940

184F2859-257C-568A-8956-C9DE852F455C

Anisembia (Chelicerca) Ross, 1940: 656, as subgenus of Anisembia Krauss, type species Anisembia (Chelicerca) davisi Ross by original designation.
Chelicerca Ross, 1944: 448; Szumik 1996: 51–52, redelimitation of genus and cladistic analysis/phylogeny; Ross 2003: 67, redescription; Szumik et al. 2008: 999–1001, redelimitation of genus and cladistic analysis-phylogeny; Miller 2009: 7, catalog; Szumik et al. 2019: 9, tympanal hearing, silk ejectors, leg chaetotaxy, phylogeny.
Chelicerca (Protrochelicerca) Ross, 1944: 449, type species ChelicercadampfiRoss; 1984b: 30, as synonym of Chelicerca Ross.
Anisembia (Dactylocerca) Ross, 1940: 659, as subgenus of Anisembia Krauss, type species Anisembia (Dactylocerca) rubra Ross by original designation; 1944: 454, as subgenus of Chelicerca.
Dactylocerca 1984a: 85, genus status; 1984b: 37, diagnosis; Szumik 1996: 51–54, as junior synonym of Chelicerca Ross; Ross 2003: 78, diagnosis, composition; Szumik et al. 2008: 999, as probable junior synonym of Chelicerca Ross; confirmed junior synonym of Chelicerca Ross.
Schizembia Ross, 1944: 440, type species SchizembiagrandisRoss; 1984a: 30, as junior synonym of ChelicercaRoss; 2003: 59, changed status; Szumik et al. 2008: 999–1001, as probable junior synonym of Chelicerca Ross; **new junior synonym of Chelicerca Ross**.
Pelorembia Ross, 1984a: 41, type species Pelorembiatumidiceps Ross; Szumik 1996: 51–54, as junior synonym of Chelicerca Ross; Ross 2003: 89, diagnosis; Szumik et al. 2008: 999, as probable junior synonym of Chelicerca Ross; confirmed junior synonym of Chelicerca Ross.
Cryptembia Ross, 2003: 49, type species Cryptembiaamazonica Ross; Szumik et al. 2008: 999–1001, as probable junior synonym of Chelicerca Ross; **new junior synonym of Chelicerca Ross**.

##### Diagnosis.

10T with completely separate hemitergites, 10Lp1 and 10Rp1 usually with discoidal form ending with a spine. H globose, Hp elongate, ending with a complex apical margin, in some cases ending in a spine-shaped lobe, in other cases ending truncated or in two small lobes; Rpp totally fused to Hp and almost inconspicuous.

##### Composition and distribution.

According to Szumik (1996), Szumik et al. (2008), and the last cladistical analysis of the Order (Szumik et al. 2019), the genera *Pelorembia*, *Dactylocerca*, *Schizembia*, and *Cryptembia* were previously treated as probable junior synonym of *Chelicerca* and here these synonymies are confirmed (see below for relationships). Thus, the genus *Chelicerca* contains 76 species distributed from the southern United States to Argentina (Szumik 1998b, 2001; Ross 2003). In Brazil six species described by Ross (2003) occur and one new species described below. Four of the species described by Ross (2003) were originally described in *Cryptembia*, here designated a new junior synonym of *Chelicerca*.

Herein we only list the seven species of *Chelicerca* present in Brazil: *Chelicercaachilata* sp. nov., see below; *Chelicercaamazonica* (Ross, 2003) comb. nov.; *Chelicercamanauara* (Ross, 2003) comb. nov.; *Chelicercaparaense* (Ross, 2003) comb. nov.; *Chelicercarioensis* Ross, 2003; *Chelicercarondonia* (Ross, 2003); *Chelicercarossi* nom. nov. because *Chelicercarondonia* Ross, 2003 is a junior primary homonym of *Cryptembiarondonia* Ross, 2003 and is transferred to *Chelicerca* (see Catalog). New locality records for *C.manaurara* are added for two states of Brazil (see Catalog).

##### Distribution.

America.

##### Relationships.

*Chelicerca* appeared as a paraphyletic group in all the phylogenetic analyses performed for the Order Embioptera (Szumik 1996; Szumik et al. 2008, 2019; Miller et al. 2012). *Pelorembia* and *Dactylocerca* appeared included in the *Chelicerca* clade and therefore were proposed as junior synonyms of *Chelicerca* by Szumik (1996). Subsequently, and by including new taxa and characters, Szumik et al. (2008) found a similar resolution for the three genera plus the inclusion of two other genera, *Schizembia* and *Cryptembia*, the five forming a monophyletic group, highly supported by some synapomorphies and almost no homoplasy. In the study of Miller et al. (2012), the three analyzed species of *Chelicerca* appeared also closely related to *Cryptembia* and *Dactylocerca*. As the genera *Schizembia* and *Cryptembia*, in addition to *Pelorembia* and *Dactylocerca*, appeared as non-monophyletic and their species as members of *Chelicerca* (Szumik et al. 2019), these four genera are being confirmed and proposed as synonymous juniors of *Chelicerca* in this work.

#### ﻿ Chelicerca achilata

Taxon classificationAnimaliaEmbiopteraAnisembiidae

Szumik, Pereyra & Juárez sp. nov.

5BABD461-92BA-5948-A470-8A5541C67F3F

http://zoobank.org/66A4ABB0-AF28-4EAC-BB0F-3AA022A6448F

Figs 1–6

##### Type material.

***Holotype***: male, Brazil: Rio de Janeiro, Nova Friburgo, Macaé de Cima, 22°22'30"S, 42°29'45"W, 14-I-2008, 1400 m, P. C. Grossi leg., Amadilha Luminosa, INPA.

##### Diagnosis.

*Chelicercaachilata* sp. nov. can be distinguished from other species of *Chelicerca* by the broader and elevate arch on inner margin of 10R, the Ep broad and clearly visible, fused to 10R; 10L also with a broad and elevated arch on inner margin, 10Lp1 with tips rounded, bilobed. LC1dp conical, well developed.

##### Description.

**Figures 1–6.**
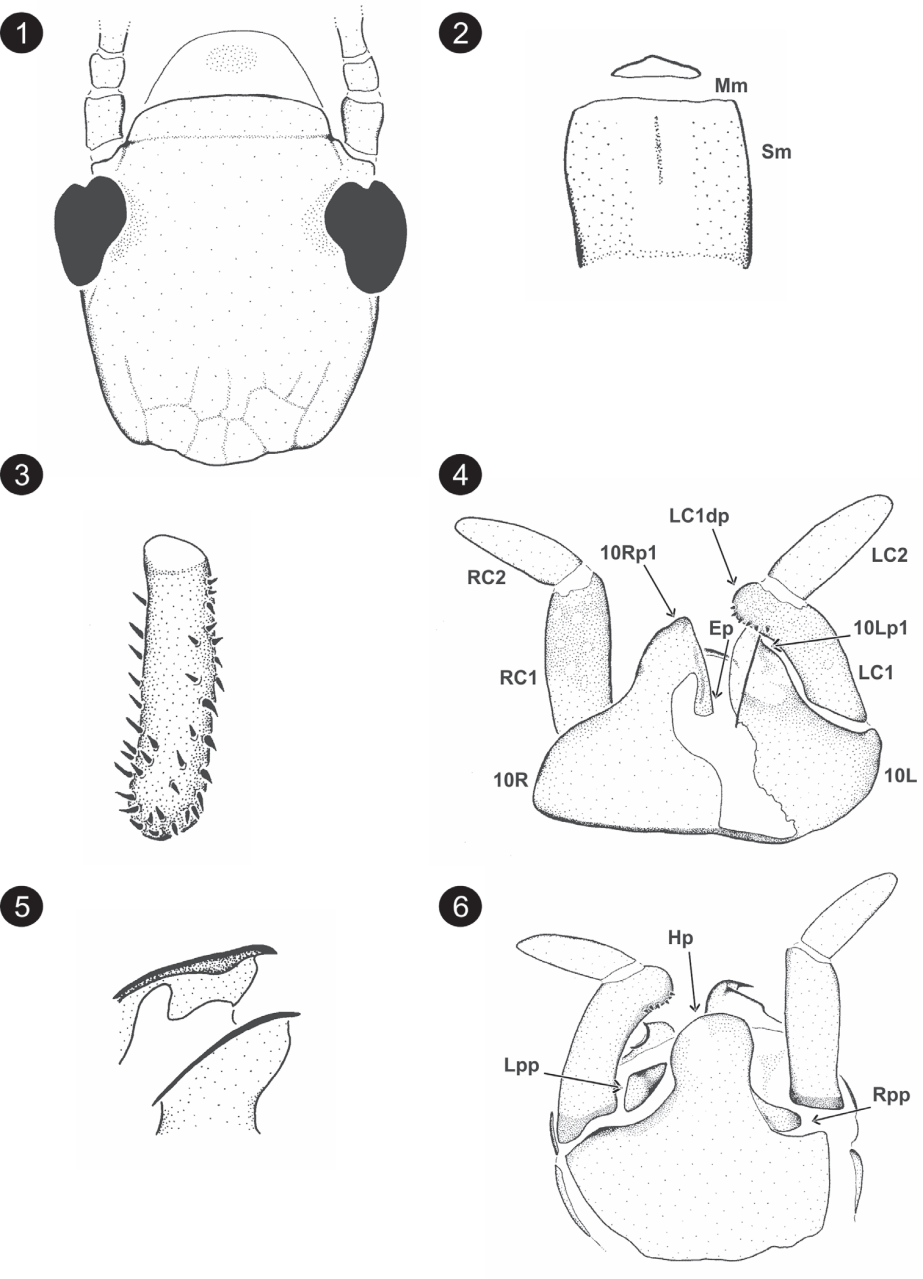
*Chelicercaachilata* Szumik, Pereyra & Juárez, sp. nov. **1** head, dorsal view **2**Mm+Sm**3** basitarsus of hind right leg **4** terminalia, dorsal view **5**10Rp1 and 10Lp1, outer lateral view **6** terminalia, ventral view.

**Male (*holotype*).** Head, prothorax, legs and terminalia light brown; antennae, pterothorax and rest of the body brownish white. Total length 6.62. Head (Fig. 1) width/length = 0.90; OR = 0.58; Md with 1–1 incisor teeth and 2–1 molar teeth; Mm conspicuous; Sm quadrangular with anterior margin membranous (Fig. 2). Forewing length 5.76, hindwing length 4.61. Wing base union type B; wing venation: Sc, R1, Rs, Cu, and A conspicuous; Ma, Mp, and Cua diffuse, less conspicuous, clearly not reaching wing edge; cross-veins in forewing: R1-Rs: 3. Basitarsus of hind leg narrow (Fig. 3): length 0.26, width/length = 0.31; 1 rows of setae on retrolateral face, single row on anterolateral face, four rows on ventrobasal face (Fig. 3). Terminalia (Figs 4–6), 10Lp1 appears oblique in reference to 10L (Fig. 4), in outer lateral view is possible to observe the two acute tips (basal and apical position, Fig. 5), the apical tip is clear to observe also on ventral view. Inner edge of 10Rp1 with a broad and prominent arch (Fig. 4). Ep well sclerotized, without microtrichia, broad and clearly fused to 10R (Fig. 4). Hp with a few transversal keels, rounded, semicircular without any additional process (Fig. 6). LC2 shorter than LC1, longitudinal ratio of LC1/LC2 = 1.09, LC1dp conical with setae; LC1dp/LC1width = 1.67. **Female.** Unknown.

##### Etymology.

Achilata is a popular homemade ice-cream in Tucumán, and is also the nickname of Claudia’s cousin, to whom this species is dedicated.

#### ﻿Genus Saussurembia

Taxon classificationAnimaliaEmbiopteraAnisembiidae

Davis, 1940

295515A8-C43C-5EE2-AA9F-83D033F59F83

Saussurella Davis, 1939b: 573, type species Embiaruficollis de Saussure, 1896 by original designation.
Saussurembia Davis, 1940a: 191, for Saussurella Bolívar, 1887 preoccupied name; Davis 1940d: 537, as a genus of Anisembiidae; Ross 1940: 647, as a genus of Mesembiinae (Anisembiidae); Ross 1944: 435, redescription of the genus; Ross 1992: 126, Saussurembiadavisi Ross as new name for Saussurembiaruficollis Davis, specimen type misidentified by Davis; Ross 2003: 15, redescription of the genus; Edgerly et al. 2007: 388, discussion of the limits of the genus; Szumik et al. 2008: 999–1001, redelimitation of the genus and cladistic analysis-phylogeny; Miller 2009: 7, 22, catalog and discussion, synonym; Szumik et al. 2019: 9, tympanal hearing, silk ejectors, leg chaetotaxy, phylogeny.
Stenembia Ross, 1972: 139, type species Stenembiaparenensis Ross, 1896 by original designation; Edgerly et al. 2007: 388, as probable junior synonym of Saussurembia Davis; Szumik et al. 2008: 999–1001, as probable junior synonym of Saussurembia Davis; **new junior synonym of Saussurembia Davis**.

##### Diagnosis.

Md acute and small, LC1 symmetrical (processes and setae absent), Hp elongate, Ep narrow (stick-like), wing venation (only veins Rs + Ma and Rs sclerotized and cross veins absent except between R1 and Rs), 10Lp1 and 10Rp1 simple, laminate narrow lobe.

##### Composition and distribution.

Given that *Stenembia* Ross, 1972 is proposed here as junior synonym of *Saussurembia* Davis, 1940 (see arguments below under phylogenetic relationships), currently the genus includes seven species: *Saussurembiadavisi* Ross, 1992 from Costa Rica, *Saussurembiaalbicauda* Ross, 1992 from Panama, *Saussurembiacalypso*Edgerly et al., 2007 from Trinidad; *Saussurembiasymmetrica* Ross, 1944 from Colombia; *Saussurembiaperenensis* (Ross, 1972) from Peru; *Saussurembiaexigua* (Ross, 1972) from Brazil and the new species described below, also from Brazil.

##### Distribution.

Central and South America.

##### Relationships.

The genus *Saussurembia* was considered closely related to *Stenembia* based on the combination of characters discussed and described by Edgerly et al. (2007) for the species *Saussurembiacalypso* which share with *Saussurembia* the presence of a sclerotized line starting from the inner margin of 10Lp1, the ventrally curved 10Rp1 and the basally broad 10Lp1 with acute apex and, with *Stenembia* the well-defined Lpp and Rpp, the dorsally curved Lpp and the directed leftward Hp (Edgerly et al. 2007). *Saussurembia* and *Stenembia* share many diagnostic characters as Md acute and small, LC1 symmetrical (processes and setae absent), Hp elongate, Ep narrow (stick-like), wing venation (Rs + Ma and Rs are the only veins sclerotized) and the general shape of 10Lp1 and 10Rp1 (with small differences in size and degree of sclerotization) (Edgerly et al. 2007). Thus, one genus is paraphyletic with respect to the other, being *Saussurembia* the autapomorphic form (Edgerly et al. 2007).

Subsequently, in the phylogenetic analysis of Embioptera (Szumik et al. 2008), the two analyzed species of *Saussurembia*, *S.davisi*, and *S.calypso*, were closely related to two species of *Stenembia*, *S.perenensis*, and *S.exigua*, both genera forming a supported clade. In the last phylogenetic analysis of the Order (Szumik et al. 2019), adding new evidence from legs (ultrastructure of chaetotaxy and the chordotonal organ), the genera continued clustered (Szumik et al. 2019: 9). The only argument used by Ross (1972: 140) when he described *Stenembia* is that the genus pertains to South America and *Saussurembia* to Central America. Because the differences between both genera are minimal, we propose *Saussurembia* Davis as senior synonym of *Stenembia* Ross.

#### ﻿ Saussurembia borba

Taxon classificationAnimaliaEmbiopteraAnisembiidae

Szumik, Pereyra & Juárez sp. nov.

27AF7350-B760-5679-B250-B0B823A606BF

http://zoobank.org/D993D11C-4EF8-4F11-ABF2-8E08F8D5FF9C

Figs 7–12

##### Type material.

***Holotype***: male, Brazil: Amazonas, Borba, Rio Abacaxis 05°15'09"S, 58°41'52"W 35 m, 27-29-V-2008, J. A. Rafael and team leg., Malaise, INPA.

##### Diagnosis.

*Saussurembiaborba* sp. nov. can be distinguished from the other species of the genus by having a strong depression on external edges of Md, basal cerci longer than apical cerci, 10Lp1 inconspicuous.

##### Description.

**Figures 7–12.**
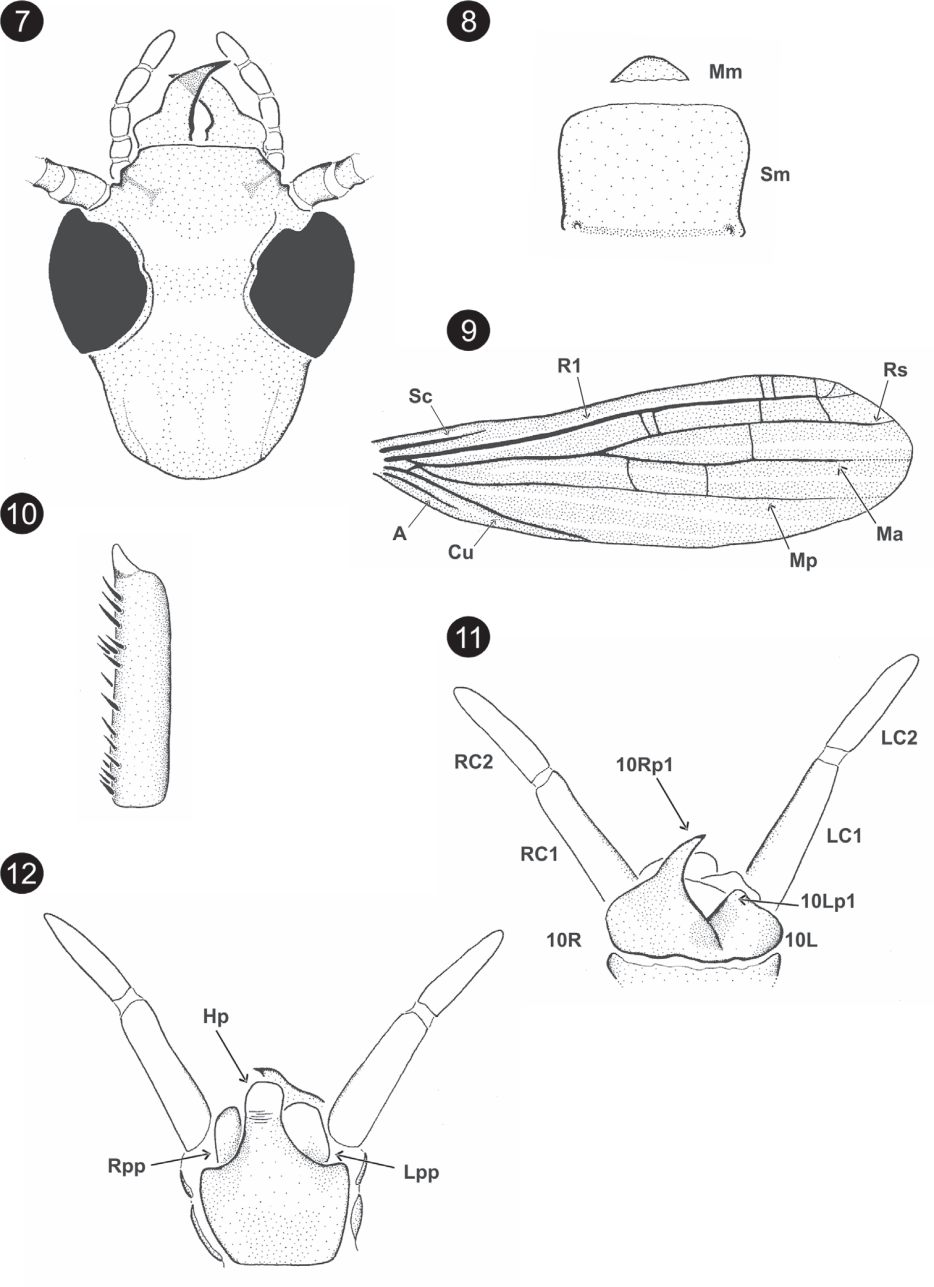
*Saussurembiaborba* Szumik, Pereyra & Juárez, sp. nov. **7** head, dorsal view **8**Mm+Sm**9** right forewing **10** basitarsus of hind right leg **11** terminalia, dorsal view **12** terminalia, ventral view.

**Male (*holotype*).** Uniformly yellowish brown with some color details: abdomen (except nine and ten segment) and cerci whitish. Total length 7.25. Head oval and elongate (Fig. 7) width/length = 0.87; anterior margin of the clypeus slightly convex, postocular suture present; eyes large OR = 0.51; Md with external edges with a strong concavity, 1–1 incisor teeth and 0–0 molar teeth (almost inconspicuous, Fig. 7); Mm broad, clearly defined, Sm with anterior margin membranous (Fig. 8). Forewing length 4.60, hindwing length 4.15. Wing base union type B, wing venation: Sc, R1, Rs, Cu, and A conspicuous; Cu not forked, Ma and Mp diffuse, less conspicuous, clearly not reaching wing edge (Fig. 9); cross-veins in forewing: R1-Rs: 4, Rs-Ma: 1–2, Ma-Mp: 2. Basitarsus of hind leg narrow (Fig. 10): length 0.37, width/length = 0.19; one row of setae on retrolateral face, one row on anterolateral face (Fig. 10). Terminalia (Figs 11, 12), 10T partially and longitudinal divided into two subequal plates, 10Lp1 inconspicuous (Fig. 11), 10Rp1 is the typical observed for this genus (Fig. 11). Hp narrow and elongate, apically depigmented, Lpp and Rpp almost subequal (Fig. 12). LC2 shorter than LC1, longitudinal ratio of LC1/LC2: 1.28 (Fig. 12). Variation. Some differences were observed between the specimens. The head of some paratypes have the sides behind eyes strongly convergent and the eyes are very large (e.g., OR = 0.34). **Female.** Unknown.

##### Etymology.

The specific name refers to the type locality, Borba a municipality in the Brazilian state of Amazonas.

##### Additional records.

Brazil • ***Paratype***: 1 male, Amazonas, Maués, Rio Abacaxis, Campina Pacamiri, 04°35'49"S, 58°13'14"W, 30-31-V-2008, J.A. Rafael and team leg., arm. Malaise, INPA; *Paratype*: 1 male, Manaus, PDBFF, XII-1986, J.A. Rafael leg., INPA; 1 male without abdomen, Res. Campina, 26-27-III-1994, coleta manual, M.F.S. Fernandez leg., INPA.

### ﻿Family Archembiidae Ross, 2001

The classification proposed by Szumik (2004) elevated Archembiinae Ross to family level with the synonymization and significant delimitation of some genera previously placed as part of the Embiidae by Ross (2001). The cladistic analysis of Szumik (2004) and subsequent phylogenetic analyses of the Order (Szumik et al. 2008, 2019) support Archembiidae as a monophyletic group. The only phylogenetic analysis where Archembiidae appears as non-monophyletic was the study of Miller et al. (2012). Although that work is valid, their analysis shows some inconsistent and unresolved clades, possibly due to the use of a small number of species, to the use of a modified subset of morphological data from the matrix of Szumik et al. (2008), or to a combination of both. They solved the non-monophyly of this group proposing a new classification; delimiting Archembiidae to two genera (*Archembia* and *Calamoclostes*) and elevating Scelembiinae to family level and including there the remainder of archembiids. In the last cladistic analysis of the Order (Szumik et al. 2019), with a broader taxon sampling and new included characters, Archembiidae as well as the three subfamilies proposed by Ross (2001) were recovered, corroborating the nomenclatural changes and their monophyly. We propose that Archembiidae includes 21 genera, three fossil genera (from India, Myanmar, and USA) and 18 modern genera (from America and Africa), accounting for a total of 86 species (including the new species described below) (Ross 2001; Szumik 2004; Engel and Grimaldi 2006; Szumik et al. 2008; Engel et al. 2011; Szumik et al. 2019). We also confirm the three subfamilies proposed by Ross (2001) of Archembiinae, Pachylembiinae, and Scelembiinae. Therefore, the genera included in the family Archembiidae Ross are: subfamily Archembiinae (three genera): *Archembia* Ross, 1971; *Calamoclostes* Enderlein, 1909; *Ecuadembia* Szumik, 2004; subfamily Pachylembiinae (four genera): *Conicercembia* Ross, 1984; *Neorhagadochir* Ross, 1944 (= *Brachypterembia* Ross, 1984); *Pachylembia* Ross, 1984; †*Sorellembia* Engel & Grimaldi, 2006; subfamily Scelembiinae (14 genera): *Ambonembia* Ross, 2001 (= *Ischnosembia* Ross, 2001); *Biguembia* Szumik, 1997; *Chirembia* Davis, 1940 (= *Navasiella* Davis, 1940); *Dolonembia* Ross, 2001; *Embolyntha* Davis, 1940 (= *Argocercembia* Ross, 2001); *Gibocercus* Szumik, 1997; †*Kumarembia* Engel & Grimaldi, 2011; †*Lithembia* Ross, 1984; *Litosembia* Ross, 2001; *Malacosembia* Ross, 2001; *Ochrembia* Ross, 2001; *Pararhagadochir* Davis, 1940; *Rhagadochir* Enderlein, 1912 (= *Scelembia* Ross, 1960); *Xiphosembia* Ross, 2001.

Here, five new species of Archembiidae from Brazil (one Archembiinae and four Scelembiinae) are described and a complete list of archembiids species and new locality records are added (see Catalog). Thus, in Brazil, the family is represented by 30 species belonging to nine genera.

#### Subfamily Archembiinae Ross, 2001

##### ﻿Genus Archembia

Taxon classificationAnimaliaEmbiopteraArchembiidae

Ross, 1971

203627CC-7A7C-5EEB-8B38-67B07761B320

Archembia Ross, 1971: 30, type species Archembialacombea Ross by original designation; Szumik 1996: 51, phylogenetic analysis; Szumik 1997: 141, phylogenetic relationships; Szumik 1998a: 34, new record for the genus in Argentina; Ross 2001: 4, diagnosis and redescription; Szumik 2004: 222, phylogenetic analysis, diagnosis and delimitation of the genus, type species Archembiakotzbaueri (Navas, 1925), senior synonym of A.lacombea; Szumik et al. 2008: 1003, phylogenetic analysis; Szumik 2012: 352, composition; Szumik et al. 2019: 22, tympanal hearing, silk ejectors, leg chaetotaxy, phylogeny.

###### Diagnosis.

*Archembia* differs from the close related genera *Calamoclostes* and *Ecuadembia* by having mandibles with incisive teeth concentrated in the apex, anterior edge of Sm diffuse; apical cerci longer than basal cerci, and medial position of LC1dp (Szumik 2004).

###### Composition and distribution.

According to Szumik (2004)*Archembia* includes six species, one is known exclusively from Bolivia, *Archembiaboliviana* Ross, 2001 and the other five originally described from Brazil, but recorded in other countries: *Archembiakotzbaueri* (Navás, 1925), *Archembiabahia* Ross, 2001, *Archembiabatesi* McLachlan, 1877 also present in Peru, *Archembiadilata* Ross, 2001 also present in Argentina, and *Archembiaparanae* Ross, 2001, exclusively from Brazil (see Catalog). Here, one new species is described from Brazil. Almost all the species of the genus were described by Ross (1971, 2001), and many of them were not illustrated. In some cases, the criteria used for the creation of a new species were differences on coloration, and a few of these species are now designated junior synonyms (*A.peruviana* Ross, 2001 and *A.lacombea* Ross, 1971; see Catalog) or transferred to another genus (*Archembiaarida* Ross, 2001 from Ecuador now *Ecuadembiaarida* (Ross); see Szumik 2004).

Thanks to the observations on Ross’s collection at CAS as well as material deposited at INPA, MZUSP, MCZ, and USNM we have a better understanding of the distribution of this genus, with one species being present in the Amazon basin (*A.batesi*), four species present in the Atlantic Forest (*A.kotzbaueri*, *A.dilata*, *A.bahia*, *A.paranae*), and a new species described from the Cerrado and Pantanal ecoregion.

###### Distribution.

South America.

###### Relationships.

Several cladistic analyses suggest that *Archembia* is a well-supported genus (Szumik 2004; Szumik et al. 2008, 2019). *Archembia* is one of the basal genera of Archembiidae. The genera *Ecuadembia* and *Calamoclostes* are the sister group of *Archembia* they share the type B vein origin, a large anal area, the straight 10Lp1 with a spatulate apex and the Ep fused to the 10Rp1 (Szumik 2004; Szumik et al. 2019); the three genera conform the subfamily Archembiinae.

##### ﻿ Archembia oruma

Taxon classificationAnimaliaEmbiopteraArchembiidae

Szumik sp. nov.

CF4A35B7-0D77-5268-B1D6-FECEC265A18B

http://zoobank.org/4AD6EBF1-7739-4A5B-959A-22EFD20B8973

Figs 13–18

###### Type material.

***Holotype***: male, Brazil: Mato Grosso: Serra do Urucum-Corumbá, 30-XI-1960, K. Lenko leg., MZUSP.

###### Diagnosis.

*Archembiaoruma* sp. nov. can be distinguished from the other species of *Archembia* by having a 10Lp1 extremely prolonged and straight, with an acute and curved apex without longitudinal carina.

###### Description.

**Figures 13–18.**
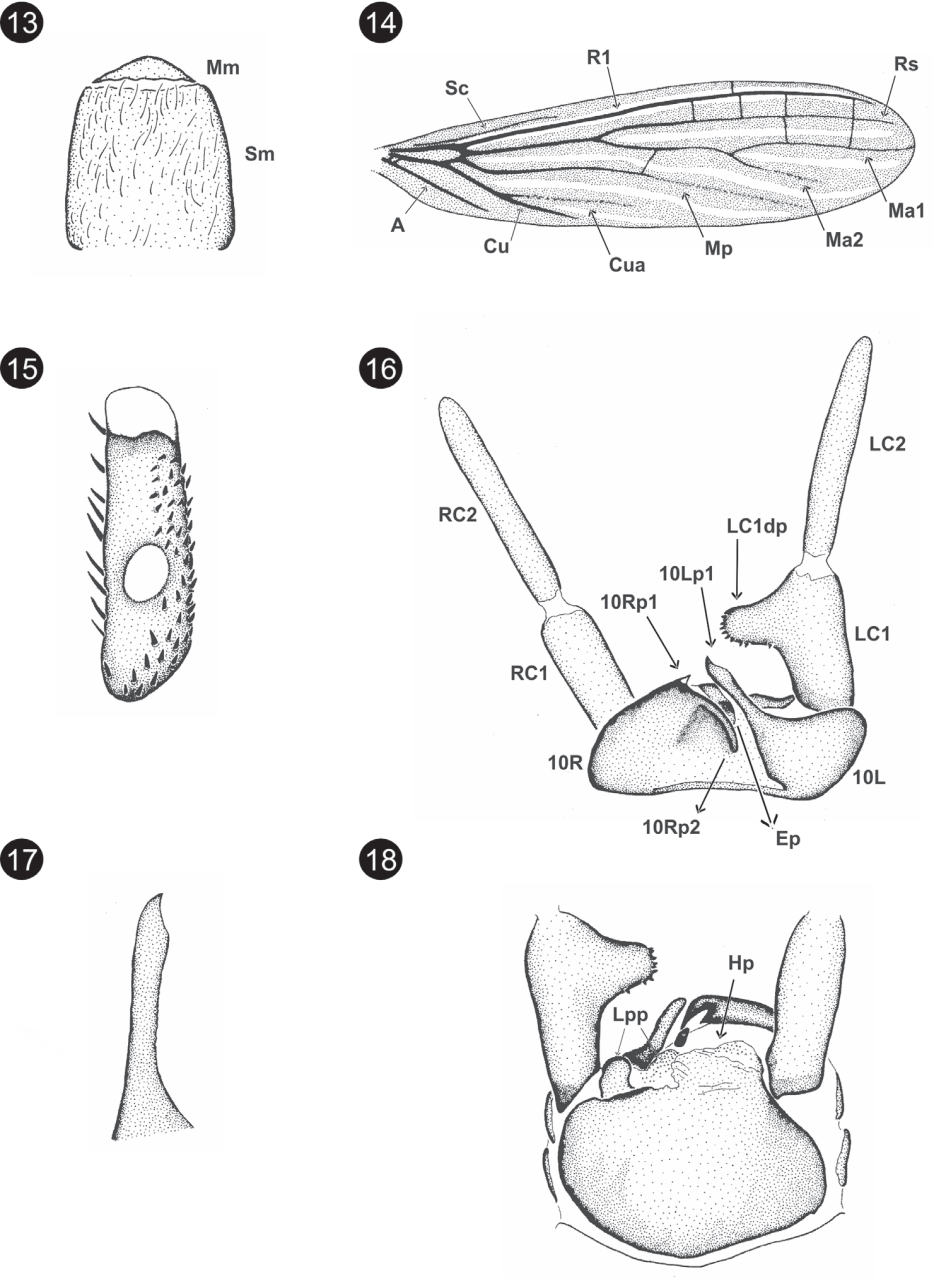
*Archembiaoruma* Szumik, sp. nov. **13**Mm+Sm**14** right forewing **15** basitarsus of hind right leg **16** terminalia, dorsal view **17**10Lp1, dorsal view **18** terminalia, ventral view.

**Male** (holotype). Uniformly orangish brown with some color details: prothorax yellowish brown and wings brown. Total length 14.00. Head quite hirsute, almost circular, postocular suture scarcely marked, width/length = 0.79; eyes not large OR = 0.60; Md: 3–2 incisor teeth and 2–1 molar teeth. Mm conspicuous, Sm hirsute, anterior margin membranous and basally broad (Fig. 13). Forewing length 9.50 mm, hindwing length 7.80. Wing base union type B, wing venation (Fig. 14): Ma, Ma1, Ma2, and Mp diffuse but clearly reach wing edge, Cua less conspicuous not reaching wing edge; cross-veins in forewing: R1-Rs: 4, Rs-Ma1: 2, Ma-Mp: 1 o 2, Cu-A: 2 (Fig. 14). Basitarsus of hind leg narrow and large (Fig. 15): length 0.58, width/length = 0.26, medial bladder large, medial bladder diameter/ basitarsus width = 0.67; single row of setae on retrolateral face, four rows on anterolateral face, two or three rows of setae on ventrobasal face. Terminalia (Figs 16–18), anterior margin of 10L slightly concave, inner basal angle of 10L excavate (Fig. 16); 10Lp1 not bifid, prolonged, and straight, without longitudinal carina, apex moderately curved (Fig. 17); 10Rp1 with apex dorsally acute and ventrally globose (Fig. 18); 10Rp2 clearly defined bar (Fig. 16). Ep conspicuous. Hp with longitudinal keels (Fig. 18), Lpp as Fig. 18, microtrichia present; Rpp not conspicuous. LC2 clearly longer than LC1, longitudinal ratio of LC1/LC2: 0.65; LC1dp medial, shape as Fig. 16. **Female.** Unknown.

###### Etymology.

The specific name is an arbitrary combination of letters.

###### Additional records.

Brazil • ***Paratype***: 1 male, same data as Holotype, MZUSP; 2 males on slides with the following labels ‘Embolynthabatesi (MacLach.)’ ‘Barro Alto, Est. Minas, Brazil Nov.’ ‘31 (José Blaser) det. Davis Proc. L.S. N.S.W., 1940 65:348’, MCZ.

#### Subfamily Scelembiinae Ross, 2001

##### ﻿Genus Embolyntha

Taxon classificationAnimaliaEmbiopteraArchembiidae

Davis, 1940

0C06867E-E886-5764-9F89-BB53222A868F

Embius ? Griffith & Pidgeon, 1832: 786, name and illustration, type species Embiusbrasilinesis Gray.
Olyntha Griffith & Pidgeon, 1832: 347, as subgenus of Embia Latreille, type species Olynthabrasiliensis Griffith and Pidgeon; Westwood 1837: 373, redescription; Burmeister 1839: 770, distinctive characters; Walker 1853: 532, catalog, diagnosis, species included; Krauss 1911: 27, erected to genus; Enderlein 1912: 29, as junior synonym of Embia; Davis 1940b: 323, 324, invalid name preoccupied by a genus of Coleoptera.
Embolyntha Davis, 1940c: 344, description and diagnosis, key to the species, type species Olynthabrasiliensis, Embius and Olyntha preoccupied names; Ross 1944: 412, discussion; Ross 1971: 29, genus restricted only to type species; Szumik 1997: 141, phylogeny; Szumik 1998a: 34, genera key; Ross 2001: 26, diagnosis; Szumik 2004: 226, diagnosis, composition, phylogeny; Szumik et al. 2008: 997, phylogeny; Miller 2009: 10, catalog; Szumik 2012: 352, list of genera from Brazil; Szumik et al. 2019: 9, tympanal hearing, silk ejectors, leg chaetotaxy, phylogeny.
Argocercembia Ross, 2001: 63, type species Argocercembiaguyana Ross; Szumik 2004: 226, junior synonym of Embolyntha Davis.

###### Diagnosis.

*Embolyntha* can be distinguished from other Archembiidae by Sm with anterior margin membranous, 10Lp1 simple and starting at inner caudal angle of 10L, 10Lp1 leaf-like with many longitudinal carinae (Szumik 2004).

###### Composition and distribution.

*Embolyntha* has two species (Szumik 2004): *Embolynthabrasiliensis* (Gray, 1832) from Brazil (without any specific location) and *Embolynthaguyana* (Ross, 2001) from Guyana and Brazil (see Catalog for details). In addition, one new species from Brazil is described below.

###### Distribution.

South America.

###### Relationships.

*Argocercembia* was synonymized with *Embolyntha* by Szumik (2004) because of the minimal differences between them. Both taxa appeared grouped together by sharing 10Lp1 shape, a small medial bladder in males, and LC1dp in a medial position, both forming a well-supported group. In the higher classification of the Order (Szumik et al. 2008: 927), the genus remains monophyletic and as sister group of the monotypic genus *Ochrembia*. Finally, these groups remain supported in a more recent study (Szumik et al. 2019: 9) where new evidence from ultrastructure traits on leg chaetotaxy as well as the chordotonal organ were included.

##### ﻿ Embolyntha oriximina

Taxon classificationAnimaliaEmbiopteraArchembiidae

Szumik, Pereyra & Juárez sp. nov.

4585F9C4-2C3A-573F-9409-602588765C89

http://zoobank.org/0605DC0D-AB88-402F-93F3-8BE70AEC3714

Figs 19–24

###### Type material.

***Holotype***: male, Brazil: Pará, Oriximiná, Rio Trombetas, Alcoa min., 16-X-1991, J. A. Rafael leg., INPA.

###### Diagnosis.

*Embolynthaoriximina* sp. nov. can be distinguished from the other species of the genus by the shape of LC1dp semispherical instead of conical (*E.brasiliensis*) or domed (*E.guyana*), absence of medial bladder on hind basitarsus, present in the other species.

###### Description.

**Figures 19–24.**
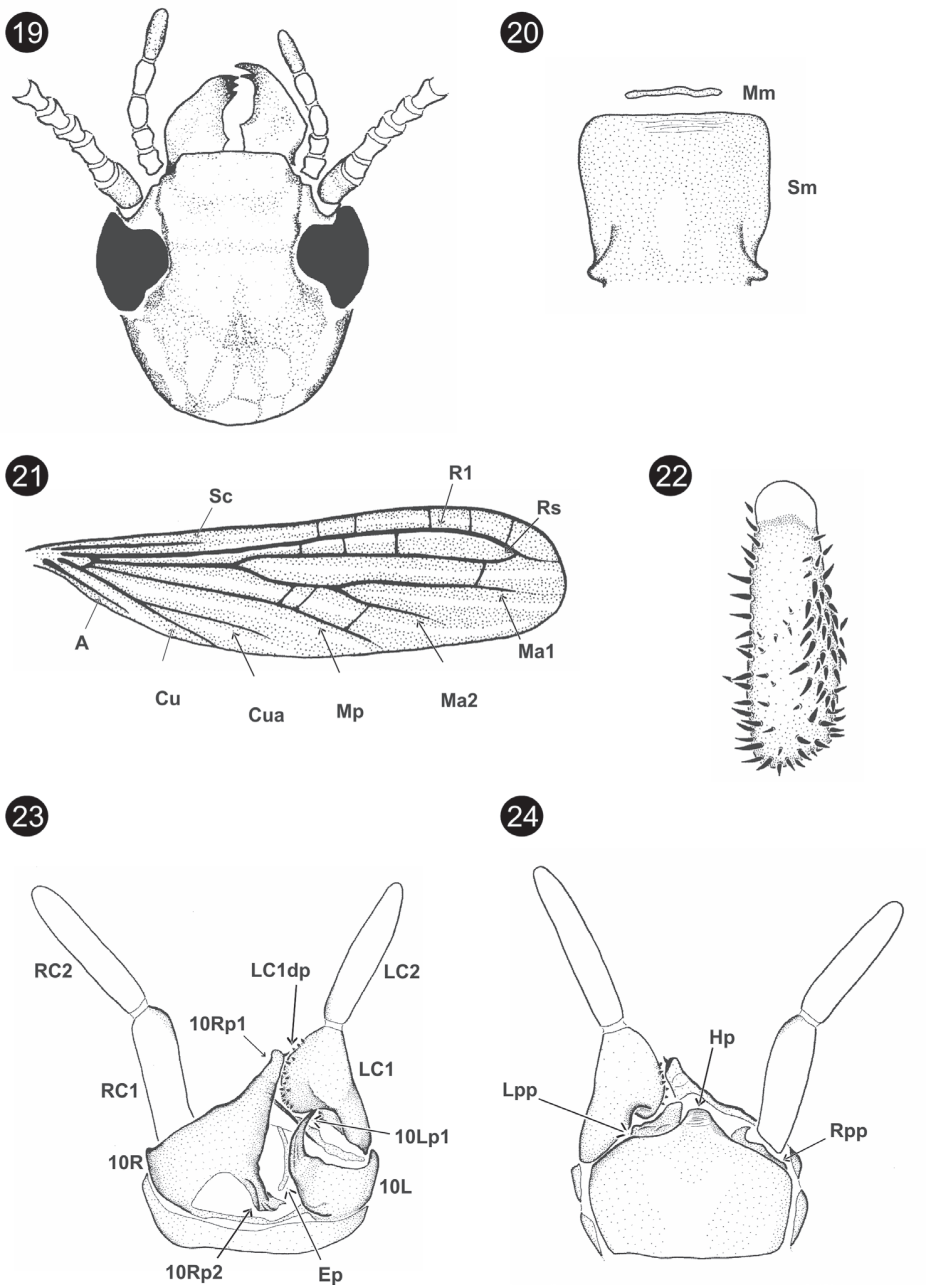
*Embolynthaoriximina* Szumik, Pereyra & Juárez, sp. nov. **19** head, dorsal view **20**Mm+Sm**21** right forewing **22** basitarsus of hind right leg **23** terminalia, dorsal view **24** terminalia, ventral view.

**Male** (holotype). General coloration brownish white with head, first antennomer, and fore tarsi brown, prothorax light brown and cerci whitish. Total length 4.00. Head (Fig. 19) width/length = 0.83; OR = 0.27; anterior margin of the clypeus straight; epistomal sulcus discontinuous; ecdysial suture as a less pigmented line quite diffuse; postocular suture as two small notches (Fig. 19); Md with 3–2 incisor teeth and 1–1 molar teeth; Mm narrow but conspicuous; Sm rectangular with anterior margin diffuse, base narrow, and surface flat (Fig. 20). Forewing (Fig. 21) length 3.82, hindwing length 3.35; wing base union type A; wing venation: Sc, R1, Rs, Ma, Ma1, Cu, and A conspicuous; Ma2 conspicuous only on the basal 1/3, Mp conspicuous only on the basal half and Cua diffuse; Ma1, Mp, and Cua clearly not reaching wing edge (Fig. 21); cross-veins in forewing: R1-Rs: 3, Rs-Ma1: 1–2; Ma-Mp: 2; Ma2-Mp: 0–1. Basitarsus of hind leg broad (Fig. 22): length 0.23, width/length = 0.39; one row of setae on retrolateral face, three rows of setae on anterolateral face, two rows of setae on ventrobasal face; medial bladder absent (Fig. 22). Terminalia (Figs 23, 24), anterior margin of 10L concave, inner basal angle of 10L excavate; 10Lp1 not bifid, acute, curved with longitudinal keels (Fig. 23); 10Rp1 bifid, on dorsal view as a broad bar and on ventral view the unsclerotized dome is observed; basal membranous area on 10R quite small, 10Rp2 broad with longitudinal keels. Ep diffuse, not well sclerotized, without microtrichia. Hp with longitudinal keels (Fig. 24), Lpp as Fig. 24. LC2 longer than LC1, longitudinal ratio of LC1/LC2 = 0.80, LC1dp as Fig. 21; LC1dp/LC1width = 2.14, LC1dp 2 × longer than the width of LC1. **Female**. Unknown.

###### Etymology.

The specific name is an apposition and refers to the type locality Oriximiná.

###### Additional records.

Brazil • ***Paratype***: 1 male, Pará, Oriximiná, Rio Trombetas, CDC, 26-IV-1981, J.A. Rafael leg., INPA; *Paratype*: 1 male, Amazonas, Manaus, Rod. AM-010, km 26, Reserva Ducke, IX-2001, J.F. Vidal leg., arm. Malaise, mata, INPA; 2 males, Pará, C. Araguaia, 19-31-I-1983, J.A. Rafael leg., INPA; 1 male, Tocantins, Xambioá, Rio Araguaia, C.D.C, 3-XI-1982, J.A. Rafael leg., INPA; 2 males, Amazonas, Rio Nhamundá, Cuipiranga, 1°53'58"S, 57°02'59"W 20-23-V-2008, JA Rafael and team leg., arm CDC, INPA.

##### ﻿Genus Pararhagadochir

Taxon classificationAnimaliaEmbiopteraArchembiidae

Davis, 1940

ED44D542-30D6-56DC-BBEB-6D7AC22DE689

Pararhagadochir Davis, 1940a: 181, type species Embiatrinitatis de Saussure by original designation; Davis 1942: 114, diagnosis; Ross 1944: 420, review; Szumik 1996: 50–51, phylogeny; Szumik 1997: 140, 141, phylogeny, relationship with other Neotropical genera; Ross 2001: 26, genera key, 43, review, diagnosis; Szumik 2004: 225, phylogeny, 230, diagnosis, composition and distribution; Szumik et al. 2008: 1003, phylogeny; Miller 2009: 11, catalog; Szumik et al. 2017: 339, as outgroup of Gibocercus Szumik and Biguembia Szumik phylogenetic analysis; Szumik et al. 2019: 9, chordotonal organ, phylogenetic analysis; Salvatierra 2020: 387, species key.

###### Diagnosis.

*Pararhagadochir* can be distinguished from other Archembiidae by having the anterior margin of Sm strongly concave, 10Lp1 apically forked with the internal tip (hook) and the external tip (flat lobe) separated, with both tips always shorter than the width of 10L. It can be differentiated by the presence of a sclerotized node between 10L and the base of LC1 and 10Rp2 with more than one longitudinal laminate keel (Szumik 2004).

###### Composition and distribution.

The genus is known from Colombia to Argentina and includes 16 species (Szumik 2004): *Pararhagadochirtrinitatis* (de Saussure, 1896) from Trinidad and Venezuela, *P.surinamensis* (Ross, 1944) from Surinam, *P.balteata* Ross, 1972, *P.bicingillata* (Enderlein, 1909), *P.castaneus* Salvatierra, 2020, *P.christae* Ross, 1972, *P.minuta* Ross, 2001, *P.noronhensis*Costa-Pinto et al., 2021 from Brazil (see Catalog), *P.flavicollis* (Enderlein, 1909), *P.tenuis* (Enderlein, 1909) from Bolivia; *P.confusa* Ross, 1944, *P.schadei* Ross, 1944, from Paraguay and Argentina; *P.birabeni* (Navas, 1918), *P.pallida* Ross, 2001, *P.trachelia* (Navas, 1915) from Argentina and *P.picchua* Ross, 2001 from Peru. Recently, *P.confusa* was also found in Brazil (Teixeira et al. 2018a). In addition, three new species from Brazil are described below and new locality records for the species *P.balteata*, *P.bicingillata*, *P.christae*, and *P.confusa* are added; the number of species of *Pararhagadochir* present in Brazil increases to ten.

###### Distribution.

South America.

###### Relationships.

*Pararhagadochir* is clearly a monophyletic genus (Szumik et al. 2008, 2019), supported by several synapomorphies (detailed in the diagnosis of the genus). The sister group is a genus from East Africa, *Chirembia*.

##### ﻿ Pararhagadochir bonita

Taxon classificationAnimaliaEmbiopteraArchembiidae

Szumik, Pereyra & Juárez sp. nov.

8D98E240-E36F-5F1A-B854-4D739F635047

http://zoobank.org/FC95AD77-A9E6-4D1E-BFDF-5F974EFD4614

Figs 25–29

###### Type material.

***Holotype***: male, Brazil: BA, Camacan, Res. Serra Bonita, 15°23'30"S, 39°33'57"W, JA Rafael and FF Xavier leg., INPA.

###### Diagnosis.

*Pararhagadochirbonita* sp. nov. differs from other species of *Pararhagadochir* by the shape of the 10Lp1 with external tip well sclerotized and the shape of LC1dp (Fig. 28).

###### Description.

**Figures 25–29.**
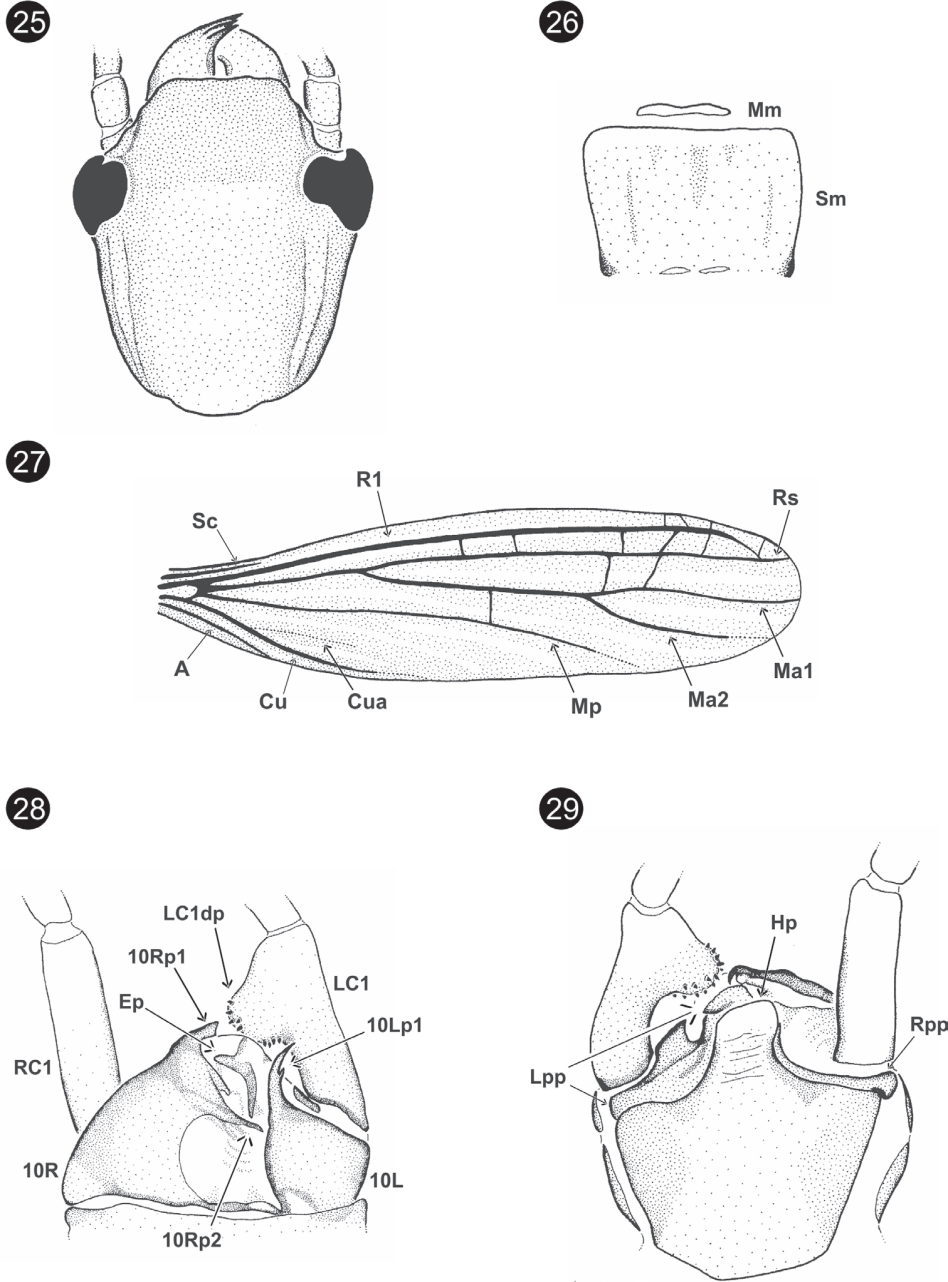
*Parahagadochirbonita* Szumik, Pereyra & Juárez, sp. nov. **25** head, dorsal view **26**Mm+Sm**27** right forewing **28** terminalia, dorsal view **29** terminalia, ventral view.

**Male** (holotype). Head, prothorax, and legs light brown, pterothorax and abdomen orangish brown, terminalia and basal cerci whitish brown, antennal tips and apical cerci white. Total length 13.21. Head (Fig. 25) width/length = 0.78; OR = 0.60; Md with 3–2 incisor teeth and 1–1 molar teeth; Mm inconspicuous, Sm with anterior margin concave, caudally constricted, surface depressed (Fig. 26). Forewing length 10.16, hindwing length 9.39. Wing base union type A; wing venation: Sc, R1, Rs, Ma, Mp, Cu, and A conspicuous (Fig. 27); Cua diffuse, Ma2 and Mp less conspicuous; Ma2, Mp, and Cua clearly not reaching wing edge; cross-veins in forewing: R1-Rs: 5, Rs-Ma1: 2–3, Ma-Mp: 1, Ma-Mp: 1. Basitarsus of hind leg no observations, hind legs lost. Terminalia (Figs 28, 29) typical of the genus but with some distinctive details: concavity at the inner basal angle of 10L present (Fig. 28); 10Lp1 base short, external tip of 10Lp1 small and strongly sclerotized (Fig. 28). Tips of 10Rp1 visible only at ventral view (Fig. 29), Ep diffuse. Hp with transversal keels (Fig. 29). Lpp with unsclerotized hook with microtrichia in the inner apical angle, close to Hp. LC2 longer than LC1, longitudinal ratio of LC1/LC2 = 0.61, LC1dp conical with setae (Figs 28, 29); LC1dp/LC1width = 1.84, LC1dp almost 2 × longer than the width of LC1. **Female.** Unknown.

###### Etymology.

The specific name refers to the type locality, Reserva Serra Bonita.

##### ﻿ Pararhagadochir marielleae

Taxon classificationAnimaliaEmbiopteraArchembiidae

Szumik, Pereyra & Juárez sp. nov.

A32D9F05-5BA2-54C2-8916-6949E73C211B

http://zoobank.org/B7368883-EB63-403A-B0E9-9D4B22693992

Figs 30–35

###### Type material.

***Holotype***: male, Brazil: MG, Serra do Caraça, 29-XI-71, Exp. Mus. Zool., MZUSP.

###### Diagnosis.

*Pararhagadochirmarielleae* sp. nov. differs from other species of *Pararhagadochir* by the shape of the 10Rp2, broad and with multiple keels, 10Lp1 base short and broad, the shape of LC1dp as Fig. 34.

###### Description.

**Figures 30–35.**
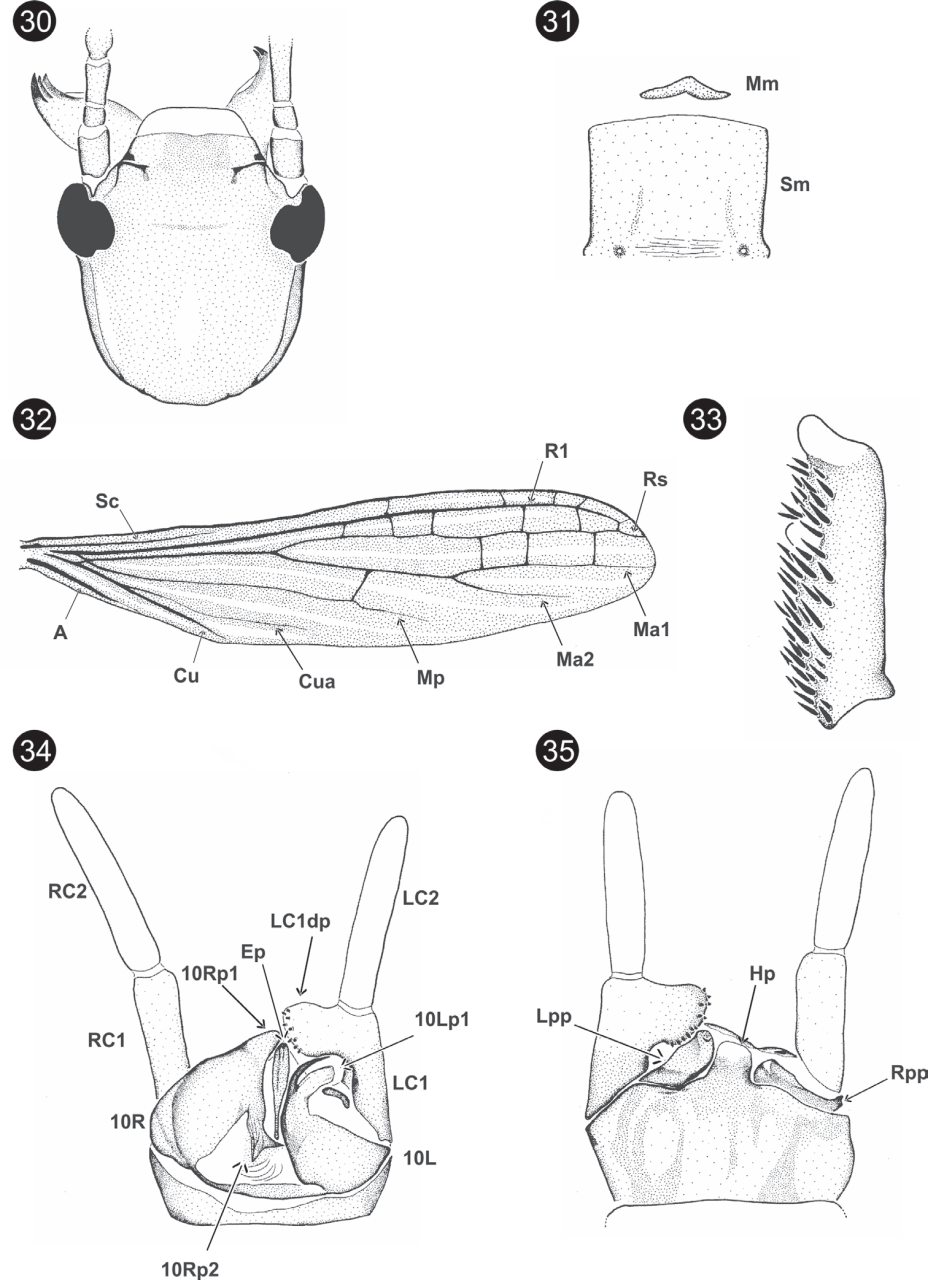
*Parahagadochirmarielleae* Szumik, Pereyra & Juárez, sp. nov. **30** head, dorsal view **31**Mm+Sm**32** right forewing **33** basitarsus of hind right leg **34** terminalia, dorsal view **35** terminalia, ventral view.

**Male** (holotype). Uniformly light brown with some color details: antenna, prothorax, and legs brownish; abdomen (except segments 9 and 10) and cerci whitish with edges of LC1dp brownish. Total length 10.18. Head width/length = 0.84, anterior margin of the clypeus slightly convex, postocular suture well developed (Fig. 30); OR = 0.60; Md with 3–2 incisor teeth and the 1–1 molar teeth, quite blunt; Mm narrow but conspicuous, Sm with anterior margin membranous, quadrangular, surface not depressed (Fig. 31). Forewing length 8.53, hindwing length 7.62. Wing base union type A; wing venation: Sc, R1, Rs, Ma, Ma1, Ma2, Mp, Cu, Cua, and A conspicuous (Fig. 32); Ma2, Mp, and Cua, clearly not reaching wing edge; cross-veins in forewing: R1-Rs: 3–6, Rs-Ma1: 2, Ma-Mp: 1. Basitarsus of hind leg narrow (Fig. 33): length 0.47, width/length = 0.27; medial bladder diameter/basitarsus width = 0.35; four rows of setae on retrolateral face, two rows on anterolateral face, five or six rows on ventrobasal face (Fig. 33). Terminalia (Figs 34, 35) typical of the genus but with some distinctive details: caudal margin of 10L straight, inner basal angle of 10L excavate (Fig. 34); 10Lp1 base short and broad, external tip of 10Lp1 broad and longer than internal tip (Fig. 34). Tips of 10Rp1 visible only at ventral view (Fig. 35), Ep apically broad, well developed. 10Rp2 apically broad with numerous longitudinal keels. Hp with transversal keels (Fig. 35) and apical edges depigmented. Lpp with unsclerotized node with microtrichia in inner apical angle, close to Hp. LC2 longer than LC1, longitudinal ratio of LC1/LC2 = 0.78, LC1dp conspicuous quadrangular with setae (Figs 34, 35); LC1dp/LC1width = 2.42, LC1dp almost 2.5 × longer than the width of LC1. **Female.** Unknown.

###### Etymology.

The specific name is a tribute to Marielle Franco, a Brazilian human rights activist, a symbol of the fight against social inequality and in favor of the rights of black women in Brazil.

##### ﻿ Pararhagadochir para

Taxon classificationAnimaliaEmbiopteraArchembiidae

Szumik, Pereyra & Juárez sp. nov.

EDF154BF-2147-5F18-9031-1A5D8BA6842F

http://zoobank.org/76AAE0BF-59E7-401E-8EA4-5193273F9F4A

Figs 36–42

###### Type material.

***Holotype***: male, Brazil: Pará, Conceição do Araguaia, 19-31-I-1983, J.A. Rafael leg., INPA.

###### Diagnosis.

*Pararhagadochirpara* sp. nov. can be distinguished by the shape of 10Lp1 (Figs 39, 40) and Lpp prominent (Fig. 42).

###### Description.

**Figures 36–42.**
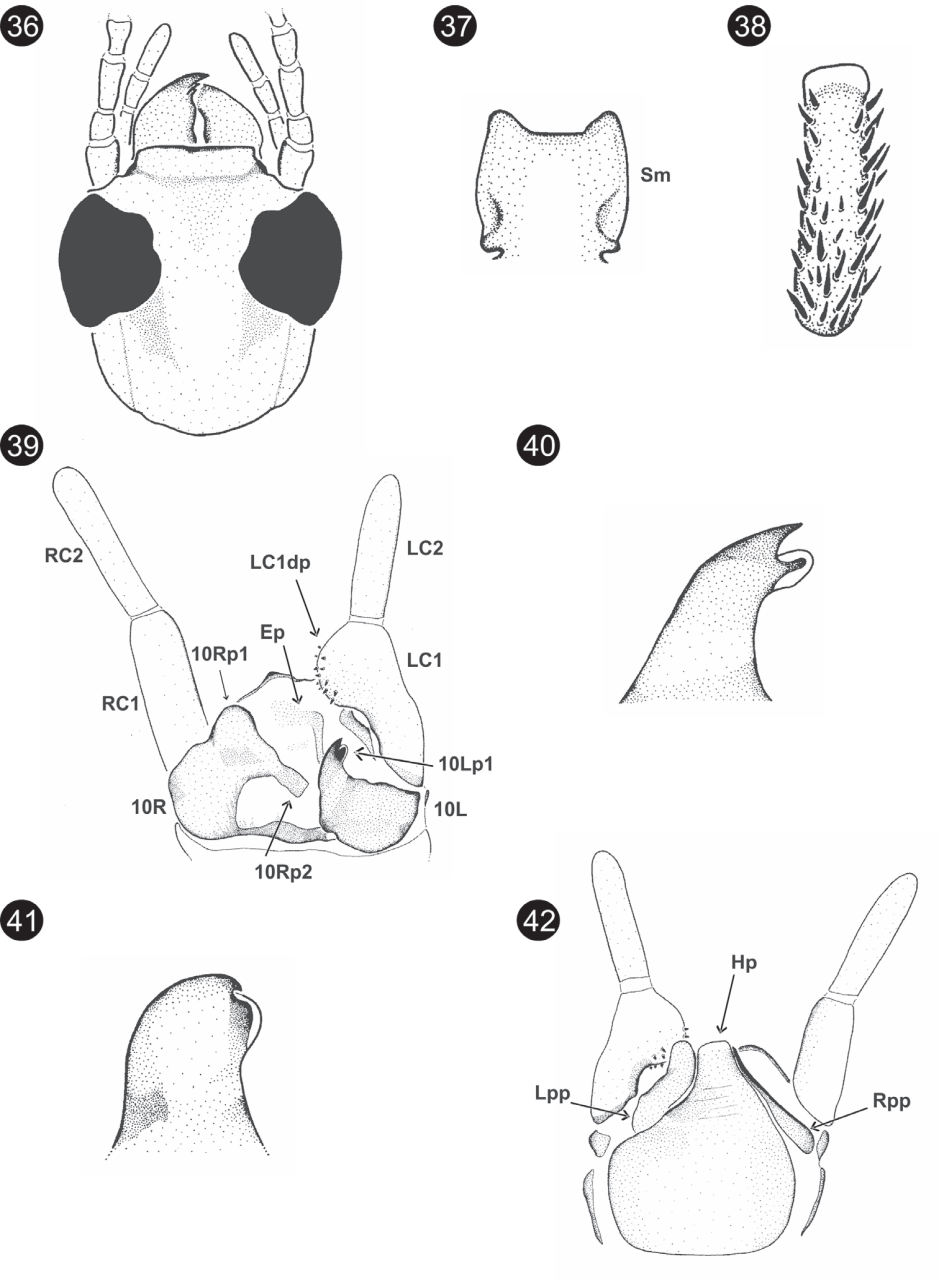
*Parahagadochirpara* Szumik, Pereyra & Juárez, sp. nov. **36** head, dorsal view **37**Sm**38** basitarsus of hind right leg **39** terminalia, dorsal view **40**10Lp1, outer lateral view **41**10Rp1, outer lateral view **42** terminalia, ventral view.

**Male** (holotype). Head dark brown, thorax and abdomen orangish brown, except cerci brownish white. Total length 5.89. Head (Fig. 36) width/length = 0.98; Anterior margin of the clypeus straight with a central small notch; epistomal sulcus discontinuous; postocular suture represented by a notch; OR = 0.32; Md with 3–2 incisor teeth and 1–1 molar teeth; Mm inconspicuous, Sm quadrangular, with anterior margin deeply concave, surface depressed (Fig. 37). Forewing length 5.10, hindwing length 4.28. Wing base union type B; wing venation: Sc, R1, Rs, Cu, and A conspicuous; Ma, Ma1, Ma2, Mp, and Cua diffuse, less conspicuous, clearly not reaching wing edge; cross-veins in forewing: R1-Rs: 3; Rs-Ma: 0–1; Rs-Ma1: 1–2. Basitarsus of hind leg narrow (Fig. 38): length 0.30, width/length = 0.24; medial bladder absent; two rows of setae on retrolateral face, one row on anterolateral face, three rows on ventrobasal face (Fig. 38). Terminalia (Figs 39–42) with the general shape present in the genus, the most striking conditions are listed here, 10Lp1 with both tips subequal and well sclerotized (Fig. 40), 10Rp1 as Fig. 41; 10Rp2 and Ep less conspicuous regarding other species of *Pararhagadochir* (Fig. 39). Lpp with a prominent inner lobe without microtrichia, Hp with keels (Fig. 42). LC2 as longer as LC1, longitudinal ratio of LC1/LC2 = 1.03, LC1dp quadrangular with setae; LC1dp/LC1width = 1.70, LC1dp almost 2 × longer than the width of LC1. **Female.** Unknown.

###### Etymology.

The specific name refers to the Brazilian state of Pará where is placed the type locality.

### ﻿Family Clothodidae Enderlein, 1909

Clothodidae is considered the most primitive family of the Order as a result of the simplicity of its morphological characters (Davis 1939a; Ross 1970, 1987; Szumik 1996). This family contains three fossil genera from Myanmar, *Atmetoclothoda* Engel & Huang, 2016 (see Engel et al. 2016); *Gnethoda* Cui & Engel, 2020 and *Henoclothoda* Cui & Engel, 2020 (see Cui et al. 2020), and five extant genera that have a mostly South American distribution with only one species from Panamá; they comprise a monophyletic group usually separated from the remaining embiids, and includes the following genera: *Antipaluria* Enderlein, 1912; *Chromatoclothoda* Ross, 1987; *Clothoda* Enderlein, 1909; *Cryptoclothoda* Ross, 1987; and *Nonaia* Engel, 2020 (see Cui et al. 2020).

Here, one new species is described for the family Clothodidae and new locality records are added. In Brazil, the family is represented by five species belonging to three genera (see Catalog).

#### ﻿Genus Chromatoclothoda

Taxon classificationAnimaliaEmbiopteraClothodidae

Ross, 1987

E0C51E01-4266-52F7-A825-5DE385B7CCBD

Chromatoclothoda Ross, 1987: 26, type species Chromatoclothodaelegantula Ross by original designation; Szumik et al. 2008: 997, cladogram; Miller 2009: 12, catalog; Szumik et al. 2019: 9, tympanal hearing, silk ejectors, leg chaetotaxy, phylogeny.

##### Diagnosis.

*Chromatoclothoda* can be distinguished from the other three genera of Clothodidae by having the male left paraproct well developed as a plate (Fig. 47), instead having both paraprocts (left and right) subequal (e.g., Ross 1987: fig. 4).

##### Composition and distribution.

*Chromatoclothoda* is a South American genus which contains five species exclusively distributed at the Amazon basin (including the new species described below): one Peruvian species, *Chromatoclothodaaurata* Ross, 1987; *C.albicauda* Ross, 1987 from Colombia and Ecuador; *C.neblina* Szumik, 2001 from Venezuela and two Brazilian species, *C.elegantula* Ross, 1987 and the new species. For *C.elegantula* new records are added (see Catalog). The Peruvian species, *C.nana* Ross, 1987 and *C.nigricauda* Ross, 1987, were recently transferred to the genus *Nonaia* (Cui et al. 2020).

##### Distribution.

South America.

##### Relationships.

*Chromatoclothoda* resulted monophyletic in two phylogenetic analyses of the Order (Szumik et al. 2008, 2019); the species of *Chromatoclothoda* share the following synapomorphies, all of which are male conditions: Md with one molar tooth; ecdysial suture inconspicuous; medial bladder size less than 40% of the width of the basitarsus; anterolateral face of hind basitarsus with one or two rows of setae; auditory organ of fore femur curved, as a slender band, elongated along the femoral axis. *Chromatoclothoda* and *Clothoda* are sister groups (Szumik et al. 2008, 2019) and are supported by sharing some “absences”, for example, the 10Lp1 is not developed and the 10Rp1 is almost inconspicuous.

#### ﻿ Chromatoclothoda langa

Taxon classificationAnimaliaEmbiopteraClothodidae

Szumik, Pereyra & Juárez sp. nov.

F5875A9D-99E5-588C-8D17-18AB868CFF1F

http://zoobank.org/F16BA508-B340-438A-875B-BE3A7572E998

Figs 43–47

##### Type material.

***Holotype***: male, Brazil: Pará, Oriximiná, Rio Trombetas-Alcoa. Min., 13-X-2000, J.A. Rafael leg., INPA.

##### Diagnosis.

*Chromatoclothodalanga* sp. nov. is close to *C.elegantula* Ross, 1987 described from specimens from Reserva Ducke, but the new species can be distinguished by several conditions: Sm uniformly depressed instead of two oval strongly depressed areas as in *C.elegantula*; Hp uniformly pigmented and starting at the right side of H instead of depigmented and starting at the center of H; 10Rp1 conspicuous, triangular instead of almost blunt as in *C.elegantula*; Ep broad and well sclerotized instead of Ep very slender as in *C.elegantula*; LC2 longer than LC1 instead of shorter than LC1 as in *C.elegantula*.

##### Description.

**Figures 43–47.**
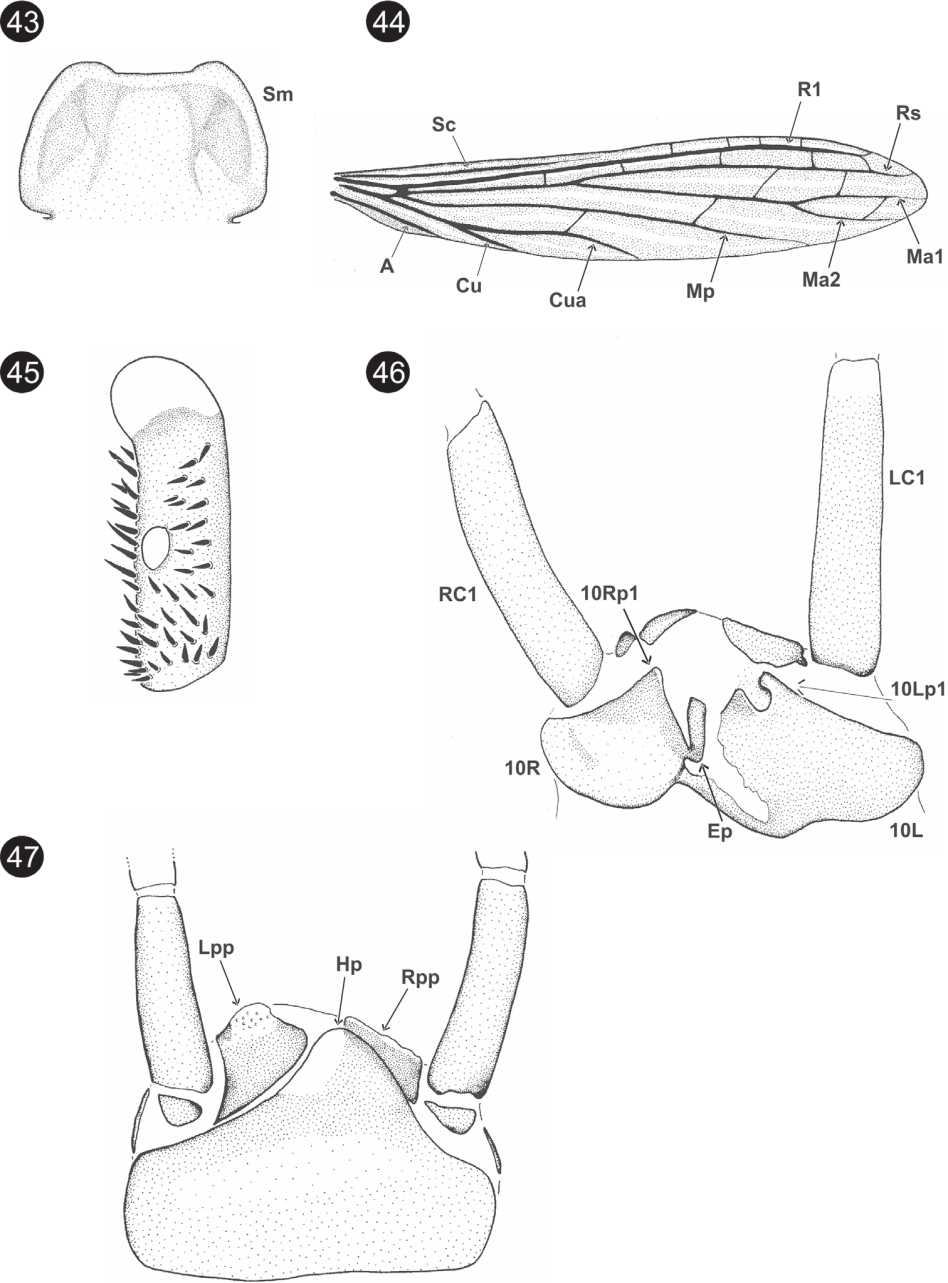
*Chromatoclothodalanga* Szumik, Pereyra & Juárez, sp. nov. **43**Sm**44** right forewing **45** basitarsus of hind right leg **46** terminalia, dorsal view **47** terminalia, ventral view.

**Male** (holotype). Head dark brown, thorax brownish yellow, antenna, legs, and abdomen brown, except for the apical antennomeres and LC2 which are white. Total length 12.22. Head width/length = 0.82, postocular suture well developed; OR = 0.62; Md with 3–2 incisor teeth and 3–2 molar teeth; Mm inconspicuous, Sm strongly depressed, base broad, wider than anterior margin, anterior margin concave (Fig. 43). Forewing length 9.12, hindwing length 8.58. Wing base union type A; wing venation: all longitudinal veins conspicuous; Ma1, Ma2, and Mp clearly not reaching wing edge, cross-veins in forewing: R1-Ma+Rs: 1; R1-Rs: 4–5; Rs-Ma: 0–1; Rs-Ma1: 1–2; Ma1-Ma2: 0–1; Ma+Rs-Mp: 0–1; Ma-Mp: 1; Mp-Cua: 1–2 (Fig. 44). Basitarsus of hind leg broad (Fig. 45): length 0.43, width/length = 0.30; medial bladder diameter/ basitarsus width = 0.31; two rows of setae on retrolateral face, two rows on anterolateral face, four or five rows on ventrobasal face (Fig. 45). Terminalia (Figs 46, 47) inner apical angle of 10L with 10Lp inconspicuous, 10Rp1 triangular and acute (Fig. 46). Membranous area between 10R and 10L narrow, Ep conspicuous. Hp not centered, starts in the right side of H (Fig. 47). Lpp is a well sclerotized plate, almost the same size as Hp; Rpp reduced as narrow well sclerotized band (Fig. 47). LC2 slightly longer than LC1, longitudinal ratio of LC1/LC2 = 0.85. **Female.** Unknown.

##### Etymology.

This species is dedicated to Langa, a childhood friend of Lucia and Victoria (Claudia’s daughters).

### ﻿Family Teratembiidae Krauss, 1911

Teratembiidae family is composed of five genera and considered a monophyletic group and sister group to Oligotomidae family (Ross 1952; Szumik 1996; Szumik et al. 2008; Miller et al. 2012; Szumik et al. 2019). Includes the genera *Diradius* Freiderichs, 1934; *Oligembia* Davis, 1939; *Teratembia* Krauss, 1911 from the American continent, *Paroligembia* Ross, 1952 from Africa, and *Dachtylembia* Poolprasert, 2014 from Thailand. In Brazil, the family is represented by seven species belonging to three genera: *Diradiuspusillus* Friederich, 1934; *D.plaumanni* (Ross, 1944); *D.unicolor* (Ross, 1944) comb. nov.; *O.bicolor* Ross, 1944; *O.versicolor* Ross, 1972; *Teratembiaproducta* Ross, 1944, and a new record reported here, *T.bancksi* Davis, 1939. New locality records are added as well as a complete list of the species known for Brazil (see Catalog).

### ﻿Catalog of Embioptera present in Brazil

The list of species occurring in Brazil (not including introduced species like *Oligotomasaundersii* Westewood; for details see Poolprasert 2012) is presented, with new combinations, new species, and new records are in bold. With a few exceptions, the holotypes listed here were observed by VP and CS; when the holotype was not available paratypes were examined, with a comment. The states and Federal District of Brazil are included as acronyms: AM, Amazonas; AP, Amapá; BA, Bahia; CE, Ceará; DF, Distrito Federal; ES, Espírito Santo; GO, Goiás; MA, Maranhão; MG, Minas Gerais; MS, Mato Gosso do Sul; MT, Mato Grosso; PA, Pará; PB, Paraíba; PE, Pernambuco; PI, Piauí; PR, Paraná; RJ, Rio de Janeiro; RO, Rondônia; RR, Roraima; RS, Rio Grande do Sul; SC, Santa Catarina; SP, São Paulo; TO, Tocantins.

#### ANISEMBIIDAE Davis, 1940

Fig. 48

***Brasilembiabeckeri* Ross, 2003**

*Brasilembiabeckeri* Ross, 2003: 59, Male Holotype, Female Allotype CAS, type locality: **Brazil**: **RJ**, Paineiras, Parque Nac. Tijuca, Rio de Janeiro.

**Additional records: Brazil: RJ**, Parque Nac. do Itatiaia, (east slope), 2100 m; **SC**, 20 km N Itajai; near Barra Velha, 50 m; 15 km W Blumenau; **PR**, Rondon, CAS.

***Chelicercaachilata* Szumik, Pereyra & Juárez, sp. nov.**

*Chelicercaachilata* Szumik, Pereyra & Juárez, sp. nov., Male Holotype INPA, type locality: **Brazil**: **RJ**, Nova Friburgo, Macaé de Cima.

***Chelicercaamazonica* (Ross, 2003) comb. nov.**

*Cryptembiaamazonica* Ross, 2003: 50, Male Holotype, Female Allotype CAS, type locality: **Brazil**: **AP**, Vila Amazonas, near Macapá.

**Additional record: Brazil**: **AP**, Vila Amazonas, near Macapá, Paratypes CAS.

***Chelicercamanauara* (Ross, 2003) comb. nov.**

*Cryptembiamanauara* Ross, 2003: 52, Male Holotype CAS, Female unknown, type locality: **Brazil**: **AM**, 10 km N Manaus.

**New records: Brazil**: **AM**, Manaus, Rod. AM-010, km 26, Reserva Ducke; Ig. Ubere; Ig. Acara, INPA.

***Chelicercaparaense* (Ross, 2003) comb. nov.**

*Cryptembiaparaense* Ross, 2003: 51, Male Holotype CAS, Female unknown, type locality: **Brazil**: **PA**, Mata da Pirelli, Marituba.

***Chelicercarioensis* Ross, 2003**

*Chelicercarioensis* Ross, 2003: 108, Male Holotype, Female Allotype MZUSP, type locality: **Brazil**: **RJ**, Rio de Janeiro. Material observed: Male and Female, Paratypes same locality as holotype CAS.

***Chelicercarondonia* (Ross, 2003) comb. nov.**

*Chriptembiarondonia* Ross, 2003: 55, Male Holotype, Female Allotype CAS, type locality: **Brazil**: **RO**, Schmidt Farm, 67 km SW Ariquemes.

***Chelicercarossi* Szumik, Pereyra & Juárez, nom. nov.**

*Chelicercarondonia* Ross, 2003: 113, Male Holotype, Female Allotype CAS, type locality: **Brazil**: **RO**, 62 km S Ariquemes, Fazenda Rancho Grande [primary junior homonym of *Chelicercarondonia* (Ross, 2003) **comb. nov**.]

***Isosembiaaequalis* (Ross, 1944)**

*Mesembiaaequalis* Ross, 1944: 438, Holotype male USNM, type data: **Brazil**: **SC**, Nova Teutonia; Mariño 1984: 91, distinguished from *Mesembiajuarenzis* Mariño; Szumik et al. 2019: 9, tympanal hearing, phylogeny.

*Isosembiaaequalis*: Ross 2003: 15, comb. nov.; Szumik et al. 2008: 1001, phylogeny; Szumik 2012: 354, list of species of Brazil.

**Additional records: Brazil**: **SC**, Nova Teutônia, Paratypes, MZUSP; Ridge immediately north of Seara, CAS.

***Oncosembiabiarmata* Ross, 2003**

*Oncosembiabiarmata* Ross, 2003: 120, Holotype Male CAS, type data: **Brazil**: **BA**, 20 km SW Jequie.

**Additional record: Brazil**: **BA**, 10 km SE of lpiau, CAS.

***Platyembiatessellata* Ross, 2003**

*Platyembiatessellata* Ross, 2003: 47, Male Holotype, Female Allotype CAS, type locality: **Peru**: Madre de Dios, Explorer’s Inn Rio Tambopata; Teixeira et al. 2018: 120, new record, **Brazil**: **RO**, close to rio Jaci Paraná, LABEI.

***Saussurembiaborba* Szumik, Pereyra & Juárez, sp. nov.**

*Saussurembiaborba* Szumik, Pereyra & Juárez, sp. nov., Male Holotype INPA, type locality: **Brazil**: **AM**, Borba, Rio Abacaxis.

***Saussurembiaexigua* (Ross, 1972) comb. nov.**

*Stenembiaexigua* Ross, 1972: 142, Male Holotype, Female Allotype CAS, type locality: **Brazil**: **PA**, Belém; Szumik et al. 2008: 1001, phylogenetic analysis; Szumik 2012: 354, list of species of Brazil; Szumik et al. 2019: 9, tympanal hearing, phylogeny.

**Figure 48.**
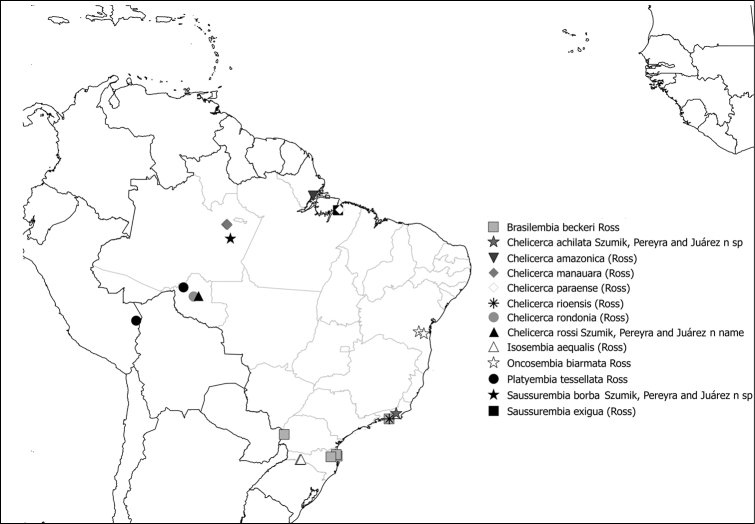
Species of Anisembiidae present in Brazil.

**Additional record: Brazil**: **PA**, Belém, Paratype, USNM.

#### ARCHEMBIIDAE Ross 2001

**ARCHEMBIINAE Ross 2001**

Fig. 49

***Archembiabahia* Ross, 2001**

*Archembiabahia* Ross, 2001: 11, Male Holotype, Female Allotype CAS, type data: **Brazil**: **BA**, on hill 20 km SW Jequié; Szumik 2004: 225, 226, phylogeny; Szumik et al. 2008: 997, phylogeny; Szumik 2012: 354, list of species of Brazil; Szumik et al. 2019: 9, tympanal hearing, phylogeny.

**Figure 49.**
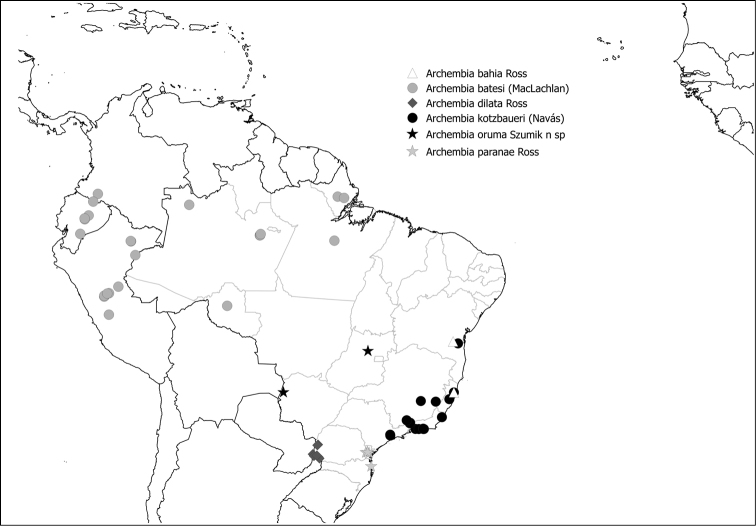
Species of Archembiinae present in Brazil.

**Additional record: Brazil**: **ES**, 20 km N of Linhares, CAS.

***Archembiabatesi* (MacLachlan, 1877)**

*Embiabatesi* MacLachlan, 1877: 380, Male Holotype NHMUK, type data: Brazil: collected by Mr. Bates in the Amazons; Navás 1918: 96, 99, species key, male redescription.

Embia (Olyntha) batesi: Hagen, 1885: 195, discussion; Kraus 1899: 148, list of Brazilian species.

*Olynthabatesi*: Krauss 1911: 29.

*Ragadochirbatesi*: Enderlein 1912: 56, comb. nov.

*Embolynthabatesi*: Davis 1940: 347, comb. nov., redescription; Barth 1954: 172, spinning apparatus; Barth and Lacombe 1955: 69, digestive system; Lacombe 1958: 177, respiratory system; 1958: 655, sexual dimorphism; 1960: 1, digestive system; 1963: 393, nervous system; 1964: 1, cephalic muscles; 1965: 503-513, Malpighian tubule system.

*Archembiabatesi*: Ross 1971: 32, comb. nov.; 2001: 6; Szumik 2004: 223, 225, phylogeny; 2012: 354, list of species of Brazil.

*Archembiaperuviana* Ross, 2001: 7 (Male Holotype CAS; Type Data: **Peru**: Huanuco, Cueva de la Pava, nr. Tingo María); Szumik 2004: 225, junior syn. of *Archembiabatesi*.

**Additional records (CAS): Brazil**: **ES**: Porto Planton; **AP**: Serra do Navio; **AM**: Vaupés (Igarapes), Rio Negro; Manaus; Médio Javari; **RO**: Rancho Grande, 62 km S Ariquemes; **Colombia**: Nariño: Macoa; **Ecuador**: Napo: Aliñahui, 25 km E Puerto Napo; Santa Rosa de Sucumbíos; Napo-Pastaza: 5 km N Puyo; Marona-Santiago: 15 km N Limon. **Peru**: Ucayali: E end Boquerón de Padre Abad; Pucallpa; Loreto: Iquitos; Amazon Camo, Rio Momón, 97.5 m (near Iquitos); Huanuco: Tingo María; 4 mi SW Las Palmas.

**New records: Brazil**: **AM**, Manaus, Campus do INPA; Reserva Ducke, INPA; **PA**, Rio Xingu Camp, 60 km S Altamira, USNM; **Ecuador**: Pastaza, Mera, CAS; **Peru**: Junin, Huacapistano, AMNH; Ucayali, Cordillera Azul, west end Boqueron de Padre Abad, CAS; 34 mi E of Tingo Maria, CAS.

***Archembiadilata* Ross, 2001**

*Archembiadilata* Ross, 2001: 12, Male Holotype CAS, type data: **Brazil**: **PR**, Foz do Iguazu; Szumik 2004: 225, 226, phylogeny; Szumik et al. 2008: 1003, phylogeny; Szumik 2012: 354, list of species of Brazil; Szumik et al. 2019: 3, tympanal hearing, silk ejectors, leg chaetotaxy, phylogeny.

**Additional record: Brazil**: **PR**, Rondon, CAS.

**New records** (FML): **Argentina**: **Misiones**, Parque Nacional Iguazú; PNI and RP101; Parque Provincial Urugua-í; Arroyo Pinalito, RP101.

***Archembiakotzbaurei* (Navás, 1925)**

*Embiakotzbaurei* Navás, 1925: 67, Male Holotype, type data: **Brazil**: **RJ**: Niterói; Davis 1939: 379, probably referable to *Clothoda* or may be listed as a species inquirenda.

*Archembiakotzbaurei*: Ross 1971: 32, type apparently lost, comb. nov., need to be redescribed; 2001: 6, spp key, 10, description, Neotype male, Neallotype female, MNRJ, Type Data: **Brazil**: **RJ**, Parque Nacional Tijuca (at Paineiras) above Rio de Janeiro, Paraneotypes CAS; Szumik 2004: 222, 224, phylogenetic analysis; Szumik 2012: 350, list of species of Brazil; Szumik et al. 2017: 339, as outgroup on phylogenetic analysis.

*Archembialacombea* Ross, 1971: 33, Male Holotype, Female Allotype CAS, Type Data: **Brazil**: **RJ**, Ponte Maromba, Parque Nacional do Itatiaia; 2001: 8, redescription, new records; Szumik, 2004: 222, 224, junior syn. of *Embiakotzbaurei* Navás, phylogenetic analysis.

**Additional records (CAS): Brazil**: **RJ**, Rio de Janeiro, Botanical Garden; Mangaratiba, SW Rio de Janeiro; Campos dos Goytacazes; Sepetiva; **ES**, Reserva Ruschi near Santa Teresa; 20 km N Linhares; **BA**,10 km SE Ipiaú; SP, Sao Paulo; **MG**, Santuario do Caraça; 20 km S Manhuaçu; Itatiaia (Natl. Park).

**New records: Brazil**: **RJ**, Cabo Frio, CAS; Paineiras, CAS; Parque Lague, MNRJ; Tijuca, CAS; MNHNP; **MG**, 24 km E Soledade de Minas, CAS; **SP**, Jardín Botánico de São Paulo, MZUSP.

***Archembiaoruma* Szumik, sp. nov.**

*Archembiaoruma* sp. nov., Male Holotype MZUSP, type data: **Brazil**: **MS**, Serra do Urucum-Corumbá.

**Additional record: Brazil**: **GO**, Barro Alto, Est. Minas, MCZ.

***Archembiaparanae* Ross, 2001**

*Archembiaparanae* Ross, 2001: 14, Male Holotype, Female Allotype MZUSP, type data: **Brazil**: **PR**, Pousada Recanto Bela Vista, picnic ground above São João da Graciosa, between Moretes and PR410, 800 m; Szumik 2004: 225, 226, phylogenetic analysis; Szumik et al. 2008: 997, phylogenetic analysis; Szumik 2012: 350, list of species of Brazil.

**Additional records (CAS): Brazil**: **PR**, Hacienda [= fazenda] Bela Vista, E of Curitiba; **SC**, N of Itajai. Material observed: Male Paratype same locality as holotype.

**New record: Brazil**: **PR**, Quatro Barra, CAS.

#### SCELEMBIINAE Ross 2001

Figs 50, 51

***Ambonembiaamazonica* (Ross, 2001)**

*Ischnosembiaamazonica* Ross, 2001: 33, Male Holotype, Female Allotype MNRJ, type locality: **Brazil**: **AP**, Vila Amazonas near Macapá.

*Ambonembiaamazonica*: Szumik 2004: 229, comb. nov., phylogeny; Szumik et al. 2008: 997, phylogeny; Szumik 2012: 354, list of species of Brazil; Szumik et al. 2019: 9, tympanal hearing, phylogeny.

**Additional record (CAS): Brazil**: **RO**, 62 km S Ariquemes. Material observed: Male Paratype same locality as Holotype.

***Biguembiacocum* Szumik, 1997**

*Biguembiacocum* Szumik, 1997: 152, Male Holotype MZUSP, type data: **Brazil: MS**, Serra do Urucum-Corumba; Ross 2001: 63, discussion; Szumik 2004: 229, phylogeny; Szumik et al. 2008: 997, phylogeny; Szumik 2012: 354, list of species of Brazil; Szumik et al. 2017: 349, phylogeny, distribution.

**Additional records (MZUSP)**: 5 Male Paratypes same data as Holotype.

***Biguembiamirador* Szumik, Gandolfo & Pereyra, 2017**

*Biguembiamirador*Szumik et al., 2017: 350, Male Holotype INPA, type data: **Brazil**: **MA**, Mirador, Parque Estadual Mirador, Base de Geraldina.

**Additional record (INPA)**: Male Paratype same data as Holotype.

***Biguembiamultivenosa* Ross, 2001**

*Biguembiamultivenosa* Ross, 2001: 61, Male Holotype, Female Allotype MZUSP, type data: **Brazil**: **PI**, 24 km SW Picos; Szumik 2004: 229, phylogeny; Szumik et al. 2008: 996, phylogeny; Szumik 2012: 354, list of species of Brazil.

**Additional records (CAS): Brazil**: **PI**, Nazaré do Piauí, ~ 42 km SE of Floriano, 84 km W of the type locality. Material observed: Male and Female Paratypes same data as Holotype.

***Biguembiaobscura* (Ross, 2001)**

*Aphanembiaobscura* Ross, 2001: 65, Male Holotype, Female Allotype CAS, type data: **Peru**: Ucayali, Yurac Plantation, 67 mi E of Tingo María.

*Biguembiaobscura*: Szumik 2004: 229, comb. nov.; Szumik et al. 2008: 997, phylogeny; Szumik 2012: 354, list of species of Brazil; Szumik et al. 2017: 351, phylogeny, distinguished from *Biguembiatroncol* Szumik.

**Additional records (CAS): Peru**: Huanuco, Tingo María; Madre de Dios, Tambopata; Loreto, Estiron, Rio Ampi Yacu; **Brazil**: **AM**, 20 km N Manaus; **RO**, Arequemes region. Material observed: Male and Female Paratypes same data as Holotype.

***Biguembiatroncol* Szumik, Gandolfo & Pereyra, 2017**

*Biguembiatroncol*Szumik et al. 2017: 351, Male Holotype INPA, type data: **Brazil**: **AM**, Itacoatiara.

***Dolonembiatapirape* Ross, 2001**

*Dolonembiatapirape* Ross, 2001: 30, Male Holotype, Female Allotype CAS, type data: **Brazil: MT**, Barra do Tapirapé; Szumik 2004: 227, diagnosis, phylogeny; Szumik et al. 2019: 9, tympanal hearing, phylogeny.

*Dolonembiataripae*: Szumik et al. 2008: 1003, phylogeny; Szumik 2012: 354, list of species of Brazil, ***lapsus calami***.

***Embolynthabrasiliensis* (Griffith & Pidgeon, 1832)**

*Embiusbrasiliensis*Griffith and Pidgeon 1832: 786.

*Olynthabrasiliensis*Griffith and Pidgeon 1832: 347, Male Holotype NHMUK, type data: **Brazil**; Westwood 1837: 373, redescripción; Burmeister 1839: 770, species list; Walker 1853: 532, catalog; Hagen 1885: 196, discussion; Krauss 1911: 28, distinctive characters.

Embia (Olyntha) brasiliensis: Krauss 1899: 148, Brazilian species and relationship with *Condylopalamaagilis* Sund. as probable junior synonym.

*Embiabrasiliensis*: Enderlein 1912: 48, redescription using other specimen from Brazil; Navás 1918: 95,98, species key, type redescription; Costa Lima 1938: 110, redescription; Davis 1940: 342, as species incorrectly referred to *Embia*.

*Embolynthabrasiliensis*: Davis 1940: 345, redescription, species type of *Embolyntha* Davis, specimen described by Enderlein 1912 as *Embiabrasiliensis* seems to be conspecific; Ross 1944: 413, discussion; Ross 1971: 29, comparisons with his new genus *Archembia*; Ross 2001: 27, type locality is formally established as **Brazil: RJ**, Paineiras, 450 m, Parque Nac. da Tijuca; Szumik 2004: 226, phylogeny; Szumik et al. 2008: 997, phylogeny; Szumik 2012: 354, list of species of Brazil.

**Figure 50.**
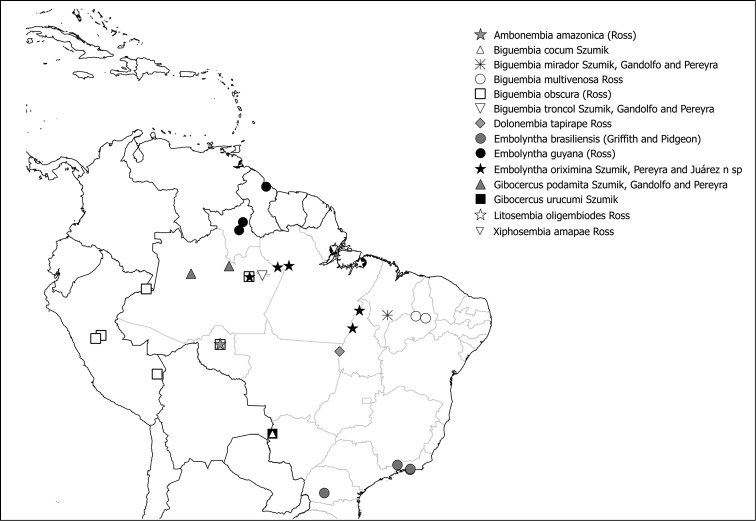
Species of Scelembiinae present in Brazil.

**Figure 51.**
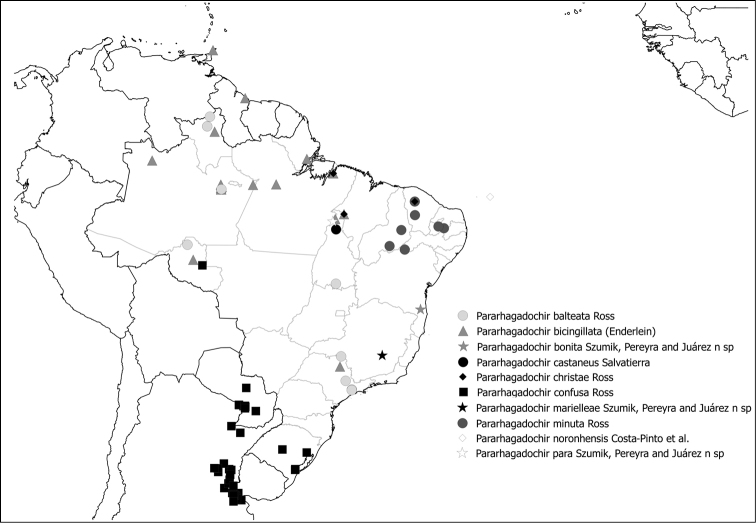
Species of *Pararhagadochir* (Scelembiinae) present in Brazil.

**Additional records: Brazil: RJ**, Rio de Janeiro, USNM; Itatiaia, CAS.

**New record: Brazil: PR**, Virmond S., ZMB.

***Embolynthaguyana* (Ross, 2001) new record**

*Argocercembiaguyana*Ross 2001: 64, Male Holotype, Female Allotype CAS, type data: **Guyana: Demerara-Mahaica**, Atkinson Airport (now Cheddi Jagan International Airport).

*Embolynthaguyana*: Szumik 2004: 227 *Argocercembia* j. syn. of *Embolyntha*; Szumik et al. 2008: 1003, phylogeny; Szumik 2012: 354, list of species of Brazil.

**New records: Brazil: AP**, Vila Amazona; **PA**, Mata da Pirelli, near Belém; **RR**, 20 km N Caracarai; Boa Vista, CAS. These materials appeared in Ross 2001 as a probable new species; these specimens were examined and we conclude that they are *E.guyana*.

***Embolynthaoriximina* Szumik, Pereyra & Juárez, sp. nov.**

*Embolynthaoriximina* Szumik, Pereyra & Juárez, sp. nov., Male Holotype INPA, type data: **Brazil: PA**, Oriximiná, Rio Trombetas.

**Additional records (INPA): Brazil: PA**, Oriximiná, Rio Trombetas; C. Araguaia; **AM**, Manaus, Rod. AM-010, km 26, Reserva Ducke; Rio Nhamundá, Cuipiranga; **TO**, Xambioá, Rio Araguaia.

***Gibocercuspodamita* Szumik, Gandolfo & Pereyra, 2017**

*Gibocercuspodamita*Szumik et al. 2017: 346, Male Holotype INPA, **Brazil: AM**, Fonte Boa, Estrada [road] Manopina.

**Additional record: Brazil: AM**, Japurá, Est. Ecol. Juami-Japurá, INPA.

***Gibocercusurucumi* Szumik, 1997**

*Gibocercusurucumi*Szumik 1997: 143–146, Male Holotype MZUSP, type data: **Brazil: MS**, Serra do Urucum-Corumba; Ross 2001: 36, discussion; Szumik 2004: 230, phylogeny; 2012: 354 list of species of Brazil; Szumik, 2017: 343, phylogeny.

***Litosembiaoligembiodes* Ross, 2001**

*Litosembiaoligembiodes*Ross 2001: 58, Male Holotype MNRJ, type Data: **Brazil: PA**, 5 km S Belém; Szumik 2004: 228, phylogeny; Szumik et al. 2008: 1003, phylogeny; Szumik 2012: 354, list of species of Brazil; Szumik et al. 2019: 9, tympanal hearing, silk ejectors, leg chaetotaxy, phylogeny.

**Additional record (CAS)**: Material observed: Male Paratypes same data as Holotype.

***Pararhagadochirbalteata* Ross, 1972**

*Pararhagadochirbalteata*Ross 1972: 133, Male Holotype CAS, type data: **Brazil: SP**, São Paulo (Park next Museu de Zoologia); Szumik 1996: 59, phylogeny; Ross 2001: 52, resdescription; Szumik 2004: 230, phylogeny; Szumik et al. 2008: 1003, phylogeny; Szumik 2012: 354, list of species of Brazil; Salvatierra 2020: 387, species key and map; Costa-Pinto et al. 2021: 144, male distinctive characters regarding *P.noronhensis*Costa-Pinto et al. 2021.

**Additional material: Brazil: SP**, São Paulo (Park next Museu de Zoologia) Paratypes USNM, MZUSP; Usina Ester, near Cosmópolis, CAS.

**New records: Brazil: AM**, Manaus, Petropolis; Conj. Tiradentes; **RO**, UHE Samuel, CDC 20 mts; **RR**, Ilha de Maracá (EE Maracá), Rio Uraricoera, INPA; Alto Alegre, Reserva Biológica Ilha de Maracá; Surumu, MZUSP; **SP**, São Paulo, Ipiranga; Buritizal, Faz. Buritiz, MZUSP; **TO**, São Salvador, INPA.

***Pararhagadochirbicingillata* (Enderlein, 1909)**

*Oligotomabicingillata* Enderlein 1909: 111, Female Holotype MZV, type data: Brazil: Para; Krauss 1911: 45, discussion; Enderlein 1912: 93, redescription; Navás 1918: 90, redescription, species key; Davis 1940: 384, unrecognizable, may belong to *Oligotomasaundersii*; Ross 1944: 497, unrecognizable species.

*Pararhagadochirbicingillata*: Ross 1972: 138, comb. nov., Male Plesiotype CAS, type data: **Brazil: PA**, Belém; 2001: 50; Szumik 2004: 230, phylogeny; Szumik 2012: 354, list of species of Brazil; Salvatierra 2020: 387, species key and map.

*Pararhagadochirdavisi*Ross 1944: 432, Male Holotype MCZ, type data: **Brazil: AM**, Parintins; 1972: 138, junior synonym of *P.bicingillata*; Ross 2001: 50, junior synonym of *P.bicingillata*.

**Additional records: Brazil: SP**, R. Preto zw Boquerao Usina Sta. Rita, NHMV; **TO**, Santa Isabel, Rio Araguaia, CAS; **AM**, Uaupés, R. Negro, CAS; Manaus, CAS; **AP**, Coracao near Macapá; **RR**, Mun. Boa Vista, Fazenda do Cabloco, CAS; **Guyana: Mahaica-Berbice**, Blairmont, CAS; **Tobago**: Canaan, Pigeon Point, CAS.

**New records: Brazil: RR**, Boa Vista, leaf litter. CAS; **MA**, 15 km S Imperatriz, CAS; **RO**, 62 km SW Ariquemes Fazenda Rancho Grande, CAS; **AM**, 10 km N Manaus, CAS; Manaus, AM-010 km 35, Sitio Vida Tropical, INPA; Manaus, INPA-II Aleixo, INPA; Manaus, INPA, Campus II, INPA; Manaus, BR 174 km 43, Est. Exp. Sil. Trop., INPA; PA, Taperinha, Santarem, MZUSP.

***Pararhagadochirbonita* Szumik, Pereyra & Juárez, sp. nov.**

*Pararhagadochirbonita* Szumik, Pereyra & Juárez, sp. nov., Male Holotype INPA, type locality: **Brazil: BA**, Camacan, Res. Serra Bonita.

***Pararhagadochircastaneus* Salvatierra, 2020**

*Pararhagadochircastaneus*Salvatierra 2020: 384, Male Holotype INPA, type locality: Brazil: **TO**, Araguaína.

***Pararhagadochirchristae* Ross, 1972**

*Pararhagadochirchristae*Ross 1972: 135, Male Holotype, Female Allotype CAS, type data: **Brazil: PA**, Belém; 2001: 51; Szumik 2004: 230, phylogeny; Szumik et al. 2008: 1003, phylogeny; Szumik 2012: 354, list of species of Brazil; Salvatierra 2020: 387, species key and map.

**Additional records: Brazil: PA**, Belém, Paratypes USNM; **CE**, near Minas do Uranio, CAS.

**New record: Brazil: MA**, 15 km S Imperatriz, CAS.

***Pararhagadochirconfusa* Ross, 1944**

*Pararhagadochirargentina* (Navás) Davis 1940a: 186, material from Paraguay, Villa Rica, MCZ; Ross, 1944: 428, erroneously identified by Davis.

*Pararhagadochirconfusa*Ross 1944: 428, Male Holotype, Paratypes, MCZ, type data: **Paraguay: Guairá**, Villa Rica; Szumik 1998: 35, on the presence of the species in Argentina; Ross 2001: 55, probably present in Paraguay, Argentina, and Brazil; Szumik 2004: 218, 222, 230, wing, male terminalia, phylogeny; Szumik et al. 2008: 1003, phylogeny; Teixeira et al. 2018: 133, new records for Brazil; Salvatierra 2020: 387, species key and map.

**Additional records: Brazil: RS**, Rio Grande do Sul; **Argentina**: **Buenos Aires**, City Zoo; **Corrientes**, CAS.

**New records: Brazil: RO**, Ariquemes, Rio Ji-Paraná, INPA; **RS**, Porto Alegre, FML; Santa Maria, MCZ; Pelotas; Capão do Leão, LABEI; **Paraguay: Central**, Asunción, FML, MNHNPA; San Lorenzo; Villeta, MNHNPA; **Concepción**, Concepción, USNM; **Guairá**, Villa Rica, MCZ; **Argentina: Buenos Aires**, Capital Federal; Coghlan; Costanera Sur, INIDEP; Ciudad Universitaria; Facultad de Veterinaria; Lago Golf, Palermo; Parque Saavedra; Adrogué; Campo de Mayo; km 26 F.C.G.B. Campo de Mayo; Cañuelas; Coghlan; Castelar; Grand Bourg; Hurlingham; Isla Martín García; La Plata; Martínez; Otamendi, INTA Delta; San Pedro; Temperley; Tigre; Rio Lujan, FML; **Chaco**, Colonia Benítez, CAS, FML; **Corrientes**, PN Mburucuyá, parcela 6; **Entre Ríos**, Arroyo Tigrecito y RN18; Arroyo Villaguay (Villaguay); Balneario La Lana; Ceibas; Crespo; 5 km Río Gualeguay; RN12 (ex R126), Arroyo Orillas del Monte; Rosario del Tala, RP39 y Arroyo Gualeguay; Villa Urquiza; **Formosa**, Clorinda; PN Pilcomayo; **Santa Fe**, Santa Fe, FML.

***Pararhagadochirmarielleae* Szumik, Pereyra & Juárez, sp. nov.**

*Pararhagadochirmarielleae* Szumik, Pereyra & Juárez, sp. nov., Male Holotype MZUSP, type locality: **Brazil: MG**, Serra do Caraça, Exp. Mus. Zool.

***Pararhagadochirminuta* Ross, 2001**

*Pararhagadochirminuta*Ross 2001: 56, Male Holotype, Female Allotype MZUSP (actually in CAS), Paratypes CAS, type data: **Brazil: CE**, 37 km NE Tauá, 425 m; Szumik 2004: 230, phylogeny; Szumik 2012: 354, list of species of Brazil; Salvatierra 2020: 387, species key and map.

**Additional records (CAS): Brazil: CE**, Mina do Uranio; **BA**, 20 km SW Casa Nova (N Bahia); **PB**, 15 km SE Patos; São Bentinho; **PI**, 15 km N Sao Raimundo Nonato, 500 m; 24 km SW Picos.

***Pararhagadochirnoronhensis* Costa-Pinto, Olivier & Rafael, 2021**

*Pararhagadochirnoronhensis*Costa-Pinto et al. 2021: 143, Male Holotype INPA, type data: **Brasil: PE**, Fernando de Noronha, Trilha Sancho.

***Pararhagadochirpara* Szumik, Pereyra & Juárez, sp. nov.**

*Pararhagadochirpara* Szumik, Pereyra & Juárez, sp. nov., Male Holotype INPA, type locality: **Brazil: PA**, Conceição do Araguaia.

***Xiphosembiaamapae* Ross, 2001**

*Xiphosembiaamapae*Ross 2001: 29, Male Holotype, Female Allotype MNRJ, Paratypes CAS, type data: **Brazil: AP**, Vila Amazonas, port of Icomi Mine, near Macapá; Szumik 2004: 227, phylogeny; Szumik et al. 2008: 2003, phylogeny; Szumik 2012: 354, list of species of Brazil; Szumik et al. 2019: 27, leg chaetotaxy, phylogeny.

**Additional record (CAS)**: Material observed: Male Paratypes same data as Holotype.

#### CLOTHODIDAE ENDERLEIN, 1909

Fig. 52

***Chromatoclothodaelegantula* Ross, 1987**

*Chromatoclothodaelegantula*Ross 1987: 27, Male Holotype, Female Allotype, Paratypes CAS, type data: **Brazil: AM**, Reserva Ducke, 10 km N Manaus; Szumik 2001: 271, comparison with *Chromatoclothodaneblina* Szumik from Venezuela; Szumik et al. 2008: 999, phylogeny; Szumik 2012: 354, list of species of Brazil.

**Additional record**: Paratypes USNM, type data: **Brazil: AM**, Reserva Ducke, 20 km Manaus.

**New Record: Brazil: AM**, Manaus, Rod. AM-010, km 36, Reserva Ducke, INPA.

***Chromatoclothodalanga* Szumik, Pereyra & Juárez, sp. nov.**

*Chromatoclothodalanga* Szumik, Pereyra & Juárez, sp. nov., Male Holotype INPA, type locality: **Brazil: PA**, Oriximiná, Rio Trombetas-Alcoa.

***Clothodanobilis* (Gerstaecker, 1888)**

*Embianobilis* Gerstaecker 1888: 1, male and female, type data: **Brazil: AM**, Itaituba; Embia (Olyntha) nobilis Kraus 1899: 148, list of species from Brazil.

*Clothodanobilis*: Enderlein 1909: 175, species type of *Clothoda* Enderlein; Enderlein 1912: 22, redescription; Navás 1918: 109, redescription of male and female; Davis, 1939: 373, Enderlein’s generic concept was based on specimens from **Brazil: AM**, Fonteboa, 374, redescription based on a specimen from Itaituba, Brazil from McLachlan Collection NHMUK, according Davis probably belongs to series from which Gerstaerker’s description was made; Ross, 1944: 406; 1987: 13 specimen of NHMUK assigned as neotype, redescription and new records; Ross 2000: 4, plesiomorphic conditions regarding fossil records, 41, female terminalia, 44, male terminalia; Szumik, 1996: 62, phylogeny; 2004: 234, outgroup on Archembiidae phylogeny; Szumik et al. 2008: 999, phylogeny; Djernæs et al. 2012: 68, outgroup sampling on Blattodea phylogeny; Kluge 2012: 381, comparison with the new species *Clothodaamazonica* Kluge from Perú; Szumik 2012: 354, list of species of Brazil; Krolow and Valadares 2016: 185, comparison with the new species *Clothodatocantinensis* Krolow and Valadares, species key; Szumik et al. 2017: 343, as outgroup on the cladistic analysis of *Gibocercus* and *Biguembia*.

**Figure 52.**
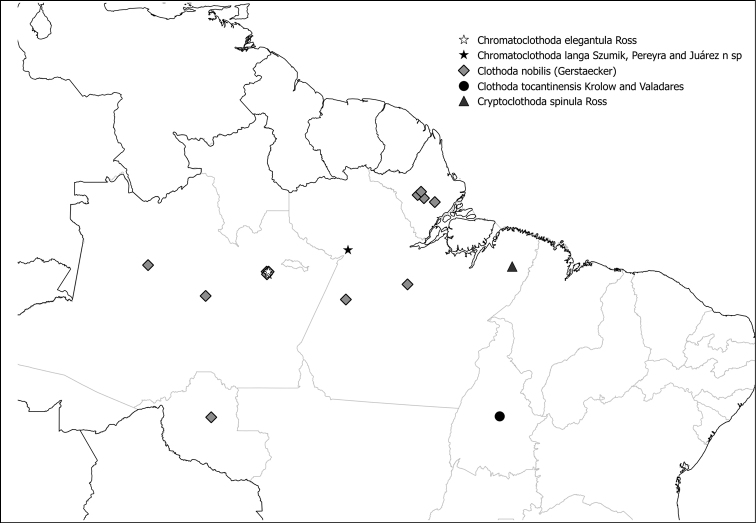
Species of Clothodidae present in Brazil.

*Olynthanobilis*: Krauss 1911: 31.

**Additional records: Brazil: AM**, Fonte Boa, MZV; Amazon basin, Reserva Ducke, 25 km N Manaus, CAS; 20 km N Manaus, CAS, USNM; Ponte da Bolivia; AP, Porto Platon; Serro do Navio; Casa do Sette, Amapari R, CAS.

**New Records: Brazil: AM**, Manaus, AM-010 km 35, Sitio Vida Tropical; Ramal Agua Branca, Sitio Vida Tropical; Coari, Lago Coari; **PA**, Medicilándia Rod. Transamazónica, Bain. Ponte de Pedra, INPA; **AP**, Amapá, MNHNP.

***Clothodatocantinensis* Krolow & Valadares, 2016**

*Clothodatocantinensis*Krolow and Valadares 2016: 185, Male holotype INPA, **Brazil: TO**, Palmas, Distrito de Taquaruçu, Fazenda Encantada, 10°15'02.3"S, 48°07'33.6"W, 07–14.XII.2012, Malaise trap, TK Krolow and HIL Lima, coll.; 2 male paratypes, same locality and collectors, 14–21.XII.2012, CEUFT and INPA.

***Cryptoclothodaspinula* Ross, 1987**

*Cryptoclothodaspinula*Ross 1987: 18, Male holotype, Female allotype CAS, type data: **Brazil: PA**, 50 km N Paragominas; Szumik 2012: 354, list of species of Brazil.

#### TERATEMBIIDAE KRAUSS, 1911

Fig. 53

***Diradiusplaumanni* (Ross, 1944)**

Oligembia (Dilobocerca) plaumanniRoss 1944: 487, Male holotype USNM, type data: **Brazil: SC**, Nova Teutonia.

*Diradiusplaumanni*: Ross 1984: 45, comb. nov.; 2000: 48, anomalous male terminalia; Szumik 1991: 612, on diagnostic characters; Szumik 1994: 71, phylogenetic analysis; Szumik 2001: 265, comparison with the new species *Diradiusnougues* Szumik; Szumik et al. 2008: 1000, phylogeny; Szumik 2012: 354, list of species of Brazil.

**Additional records: Brazil: SC**, Nova Teutonia, Paratypes CAS, USNM, MNHNP, MZUSP.

**New records: Brazil: SP**, Providencias, MZUSP; **Paraguay**, MCZ; **Argentina: Misiones**, Parque Nac. Iguazú, laboratorio; Parque Nac. Iguazú, escuela; Parque Nac. Iguazú, RP101: **Corrientes**, Parque Nac. Mburucuya, FML.

***Diradiuspusillus* Friederichs, 1934**

*Diradiuspusillus*Friederichs 1934: 419, Male holotype ZMH, type data: **Brazil: SC**, Isabelle, Humboldt Region; Davis 1940: 528, redescription; Ross 1944: 493, discussion; De Santis and De Sureda: 62 erroneous ID of a male from Argentina: Buenos Aires, Bella Vista, correct ID *Diradiusnougues* Szumik; Szumik 2012: 354, list of species of Brazil.

**Figure 53.**
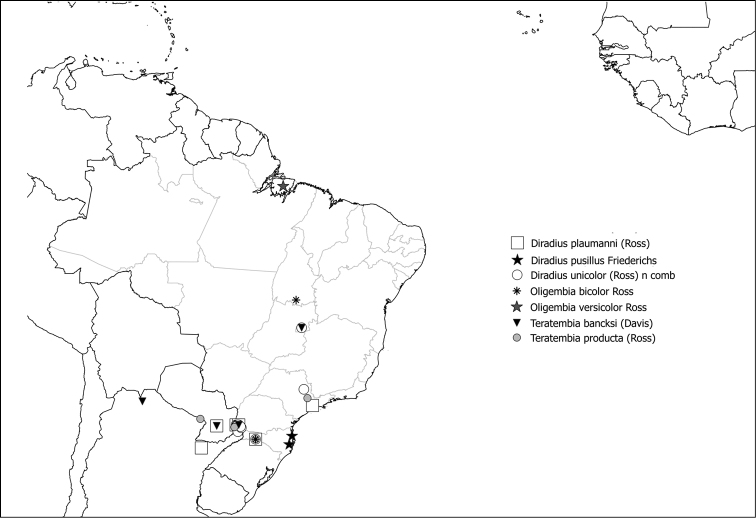
Species of Teratembiidae present in Brazil.

**New Record: Brazil: SC**, 5 mi N. Itajaí, MNHNP.

***Diradiusunicolor* (Ross, 1944) comb. nov.**

*Oligembiaunicolor*Ross 1944: 469, Male holotype CAS, type data: **Brazil: SC**, Nova Teutonia; Szumik 1991: 612, 618, redescription, comparison, and discussion regarding the new species *Diradiuserba* Szumik; Szumik 2012: 354, list of species of Brazil.

**Additional record: Brazil: SC**, Nova Teutonia, Paratypes, FML, MZUSP, MNHNP.

**New records: Brazil: DF**, Planaltina, USNM; **SP**, Est. Exp. Pirassununga, MZUSP; **Argentina: Misiones**, 44 km E de El Dorado, RP17; A° Piñalito, 2 km río abajo de RP101; Parque Nac. Iguazú, laboratorio; Parque Nac. Iguazú, RP101; Parque Nac. Iguazú, RP101, 10 km del cruce; Parque Nac. Iguazú, RP101, ca. de El Palmital; Parque Prov. Urugua-i, FML.

***Oligembiabicolor* Ross, 1944**

*Oligembiabicolor*Ross 1944: 468, Male holotype USNM, type data: **Brazil: SC**, Nova Teutonia; Szumik 1991: 618, 620, redescription, comparison, and discussion regarding the new species *Oligembiamini* Szumik; Szumik et al. 2008: 997, phylogeny; Szumik 2012: 354, list of species of Brazil.

**Additional records: Brazil: SC**, Nova Teutonia, paratypes CAS, USNM, MCZ, MZUSP, MNHNP.

**New records: Brazil: TO**, Ig. Sao Salvador, INPA.

***Oligembiaversicolor* Ross, 1972**

*Oligembiaversicolor*Ross 1972:144, Male holotype, Female allotype CAS, type data: **Brazil: PA**, Ilha Marajozinho; Szumik et al. 2008: 997, phylogeny; Szumik 2012: 354, list of species of Brazil.

**Additional record: Brazil: PA**, Ilha Marajozinho Paratypes USNM.

***Teratembiabancksi* (Davis, 1939) new record**

*Oligembiabanksi* Davis 1939: 221, Male holotype MCZ, type data: **Paraguay: Guairá**, Villa Rica.

*Idioembiabanksi*: Ross 1944: 455, comb. nov.

*Teratembiabanksi*: Ross 1952: 227, comb. nov.; Szumik et al. 2008: 997, phylogeny.

**Additional records: Paraguay: Guairá**, Villa Rica, paratype MCZ.

**New records: Brazil: DF**, Planaltina, USNM; **Argentina: Misiones**, PN Iguazú, RP101; **Salta**, La Quena, RP34, FML.

***Teratembiaproducta* (Ross, 1944)**

*Idioembiaproducta*Ross 1944: 456, Male Holotype USNM, type data: **Brazil: SC**, Nova Teutonia.

*Teratembiaproducta*: Ross, 1952: 227, comb. nov., comparison with *T.geniculata* Krauss; Szumik et al. 2008: 997, phylogeny; Szumik 2012: 354, list of species of Brazil.

**Additional records: Brazil: SC**, Nova Teutonia, Paratypes CAS, MCZ, USNM, MZUSP.

**New records: Brazil: SP**, Campinas USNM; Rio Grande do Sul, Porto Alegre FML; **Argentina: Misiones**, Parque Nac. Iguazú, escuela; Parque Nac. Iguazú, laboratorio; Puerto Libertad; **Formosa**, Parque Nac. Pilcomayo, Estero Poi, FML.

## Supplementary Material

XML Treatment for Chelicerca

XML Treatment for Chelicerca achilata

XML Treatment for Saussurembia

XML Treatment for Saussurembia borba

XML Treatment for Archembia

XML Treatment for Archembia oruma

XML Treatment for Embolyntha

XML Treatment for Embolyntha oriximina

XML Treatment for Pararhagadochir

XML Treatment for Pararhagadochir bonita

XML Treatment for Pararhagadochir marielleae

XML Treatment for Pararhagadochir para

XML Treatment for Chromatoclothoda

XML Treatment for Chromatoclothoda langa
